# The rise and fall of major royal jelly proteins during a honeybee (*Apis mellifera*) workers' life

**DOI:** 10.1002/ece3.5429

**Published:** 2019-07-05

**Authors:** Dirk Dobritzsch, Denise Aumer, Matthew Fuszard, Silvio Erler, Anja Buttstedt

**Affiliations:** ^1^ Institut für Biochemie und Biotechnologie, Pflanzenbiochemie Martin‐Luther‐Universität Halle‐Wittenberg Halle (Saale) Germany; ^2^ Proteinzentrum Charles Tanford, Core Facility ‐ Proteomic Mass Spectrometry Martin‐Luther‐Universität Halle‐Wittenberg Halle (Saale) Germany; ^3^ Institut für Biologie Molekulare Ökologie Martin‐Luther‐Universität Halle‐Wittenberg Halle (Saale) Germany; ^4^ B CUBE ‐ Center for Molecular Bioengineering Technische Universität Dresden Dresden Germany

**Keywords:** apalbumin, division of labor, gene expression, MRJP, nutrition, phenotypic plasticity

## Abstract

The genome of the western honeybee (*Apis mellifera*) harbors nine transcribed *major royal jelly protein* genes (*mrjp1‐9*) which originate from a single‐copy precursor via gene duplication. The first MRJP was identified in royal jelly, a secretion of the bees' hypopharyngeal glands that is used by young worker bees, called nurses, to feed developing larvae. Thus, MRJPs are frequently assumed to mainly have functions for developing bee larvae and to be expressed in the food glands of nurse bees. In‐depth knowledge on caste‐ and age‐specific role and abundance of MRJPs is missing. We here show, using combined quantitative real‐time PCR with quantitative mass spectrometry, that expression and protein amount of *mrjp1‐5* and *mrjp7* show an age‐dependent pattern in worker's hypopharyngeal glands as well as in brains, albeit lower relative abundance in brains than in glands. Expression increases after hatching until the nurse bee period and is followed by a decrease in older workers that forage for plant products. *Mrjp6* expression deviates considerably from the expression profiles of the other *mrjps*, does not significantly vary in the brain, and shows its highest expression in the hypopharyngeal glands during the forager period. Furthermore, it is the only *mrjp* of which transcript abundance does not correlate with protein amount. *Mrjp8* and *mrjp9* show, compared to the other *mrjps,* a very low expression in both tissues. Albeit *mrjp8* mRNA was detected via qPCR, the protein was not quantified in any of the tissues. Due to the occurrence of MRJP8 and MRJP9 in other body parts of the bees, for example, the venom gland, they might not have a hypopharyngeal gland‐ or brain‐specific function but rather functions in other tissues. Thus, *mrjp1‐7* but not *mrjp8* and *mrjp9* might be involved in the regulation of phenotypic plasticity and age polyethism in worker honeybees.

## 1 INTRODUCTION

**Figure 1:**
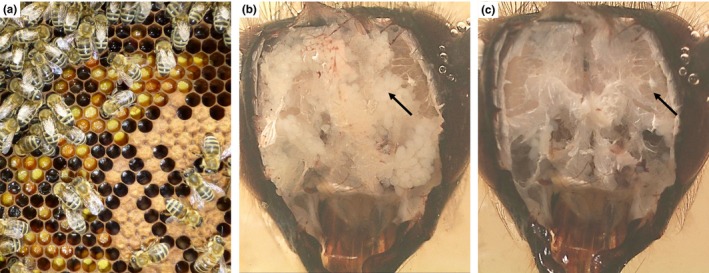
Details of worker honeybees (*Apis mellifera*) (a) Worker bees storing pollen and honey (upper left) on a frame next to nurse bees taking care of the brood (lower right). (b) Upon opening the head capsule of a bee, the hypopharyngeal glands (arrow) are visible as oval acini attached to a collecting duct. (c) After removal of the hypopharyngeal glands, the brain (arrow) can be seen below, surrounded by air sacs

Honeybee (*Apis mellifera*) workers (Figure 1a) show an elaborate age polyethism with younger workers (days 2–11, nurse bees) feeding the growing larvae and older workers (>20 days, forager bees) leaving the hive foraging for pollen, nectar, propolis, and water (Rösch, 1925; Seeley, 1982). Nurse bees feed the larvae with a special food jelly consisting mainly of water, sugars, fatty acids, and proteins (von Planta, 1888, 1889; Swammerdam, 1738). The food jelly explicitly given to larvae that develop into queens is called royal jelly (Huber, 1792). Food jelly is a composite product of fatty acids produced in mandibular glands (MGs) and proteins secreted by hypopharyngeal glands (HGs) (Callow, Johnston, & Simpson, 1959; Kratky, 1931; Patel, Haydak, & Gochnauer, 1960; Schiemenz, 1883). The main protein of this food jelly was first isolated from royal jelly in 1992 and termed major royal jelly protein (MRJP) (Hanes & Šimúth, 1992), later renamed into MRJP1. The release of the *A. mellifera* genome revealed that *mrjps* are in fact members of a multigene family that consists of nine transcribed *mrjps* (*mrjp1‐9*) and a nontranscribed pseudogene *mrjp‐ψ/10* (Drapeau, Albert, Kucharski, Prusko, & Maleszka, 2006; Helbing, Lattorff, Moritz, & Buttstedt, 2017).

All MRJPs can be detected in food jelly, with MRJP1‐3 and MRJP5 accounting for 82%–90% of total food jelly proteins (Schmitzová et al., 1998; Schönleben, Sickmann, Mueller, & Reinders, 2007; Zhang et al., 2014), and have undoubtedly a nutritional function. However, functions go far beyond that: An oligomeric form of MRJP1 (oligoMRJP1) builds a pH‐dependent fibrillary network (Buttstedt et al., 2018) in complex with apisimin, a serine–valine‐rich small protein that is another proteinaceous component of royal jelly (Bíliková et al., 2002). This fibrillary network confers the needed viscosity to royal jelly to prevent queen larvae falling out of their vertically oriented queen cells (Buttstedt et al., 2018; Kurth, Kretschmar, & Buttstedt, 2019). In addition, the complex of oligoMRJP1/apisimin binds 24‐methylenecholesterol and provides the developing larvae with essential sterols (Tian et al., 2018). MRJP3 binds and stabilizes RNA in royal jelly and is thought to share RNA among individuals (Maori et al., 2019). When worker bees were fed with labeled RNA, this RNA was found bound to MRJP3 in the food jelly produced by these worker bees (Maori et al., 2019). It is thought that this transmission of RNA from workers to larvae could drive social immunity against pathogens (Maori et al., 2019). In addition, oligoMRJP1/apisimin, MRJP2, and MRJP4 have antibacterial activity in vitro (Bíliková, Wu, & Šimúth, 2001; Kim et al., 2019; Vezeteu, Bobiş, Moritz, & Buttstedt, 2017).

Besides their expression in food‐producing HGs, *mrjp* mRNA and the resulting proteins have been detected in a variety of tissues, for example, antennae, brain, nerve chord, hemolymph, and the Malpighian tubule system, not only in worker bees but also in drones and queens (Buttstedt, Moritz, & Erler, 2014; Chan et al., 2013; Whitfield et al., 2002). However, the focus of the expression of *mrjp1‐7* was clearly assigned to the heads of worker bees (Buttstedt, Moritz, & Erler, 2013). Expression of these genes is not only upregulated in food jelly‐producing nurse bees but also in forager bees when compared to caged worker bees outside of the hive context (Buttstedt et al., 2013). Surprisingly, for *mrjp1*, *mrjp2*, *mrjp5,* and *mrjp7* expression in nurse bees was not significantly higher than in forager bees (Buttstedt et al., 2013) albeit nurse bees are feeding larvae whereas foragers do not. This partially contrasts earlier studies reporting on higher expression of *mrjp1*, *mrjp3,* and *mrjp4* in nurse bee heads (Klaudiny, Kulifajová, Crailsheim, & Šimúth, 1994; Ohashi, Natori, & Kubo, 1997) and MRJP1‐3 to only be detectable in HGs of nurse but not forager bees (Kubo et al., 1996). Apart from their occurrence in the HGs, transcripts of all *mrjps* were found in the honeybee brain in a study analyzing brain expressed sequence tag libraries (Whitfield et al., 2002) and the question arises whether the observed expression in forager heads might be caused by a shift of the expression from the HGs to the brain while the bees age.

To obtain a deeper understanding of the potential involvement of MRJPs in the appearance of phenotypic plasticity and age polyethism in honeybees, we combined quantitative real‐time PCR (qPCR) with quantitative mass spectrometry, to elucidate the intensity and timing of *mrjp* transcription and translation in both HGs (Figure 1b) and brains (Figure 1c) throughout a worker honeybee's life from hatching to the forager stage using a fully active bee hive.

## 2 MATERIALS AND METHODS

### 2.1 Honeybee samples

Honeybees (*Apis mellifera*) were sampled in May and June 2016 from a queen‐right brood‐rearing colony located in Halle (Saale), Germany (latitude: 51.5046; longitude: 11.9493). To raise age‐matched worker bees, a brood frame containing pupae with dark eyes was removed from the hive and incubated at 34°C and 60% relative humidity. A total of 600 freshly hatched bees were paint‐marked (Edding 751 gloss paint markers, Edding) on their thoraces and returned to the hive. After 0 (directly after hatching), 4, 8, 12 (nurse bee period), 16, 20 (transition phase), and 24 days (foragers), ten bees per day were freeze‐killed in liquid nitrogen and stored at −80°C until further processing. Bees were only sampled according to age, and it is not known whether, for example, 24‐day‐old bees foraged for pollen, nectar, water, or resin.

### 2.2 Gene expression

Honeybee HGs (Figure 1b) and brains (Figure 1c) of ten bees per time point were dissected, washed in insect saline (Carreck et al., 2013), and immediately placed into 200 µl buffer RA1 supplemented with β‐mercaptoethanol (NucleoSpin^®^ RNA Kit, Macherey‐Nagel). RNA was further extracted according to the manufacturer's protocol that included a DNase digestion step. The flow‐through after binding of the RNA to the NucleoSpin^®^ RNA columns was retained for subsequent protein isolation (see Section 2.3). Quantity of total RNA was photometrically determined with a NanoDrop 1,000 (Thermo Fisher Scientific), and total RNA per HG pair or brain per bee was calculated (Figure S1).

Five hundred nanogram total RNA was reverse‐transcribed using 0.4 μg oligo (dT)_15_ primer (Promega), 0.8 μl dNTPs (10 mM), and 80 U M‐MLV Reverse Transcriptase (Promega). cDNA was purified with the QIAquick PCR Purification Kit (Qiagen) as described in the manufacturer's protocol, and the concentration of each sample was diluted to 15 ng/μl. To minimize sample variation for quantitative real‐time PCR (qPCR), cDNA from three individuals was pooled, and finally, three pools per day and tissue were analyzed. qPCRs were performed as described earlier (Buttstedt et al., 2013) in a CFX Connect™ Real‐Time PCR Detection System (Bio‐Rad). Gene‐specific primers were either designed to span at least one intron using Primer‐BLAST of the National Center for Biotechnology Information (NCBI) or adopted from existing publications (Table S1) (Buttstedt et al., 2013; Evans, 2006; Lourenço, Mackert, Cristino, & Simões, 2008; Winkler, Sieg, & Buttstedt, 2018). Initially, *arp1* (*actin‐related protein 1*), *rpS5α* (*ribosomal protein S5α*), *rp49* (*ribosomal protein 49*), *pros26* (*proteasome subunit beta type‐1*), and *ppil2* (*peptidyl‐prolyl cis‐trans isomerase‐like 2*) were tested for eligibility as reference genes. Any gene with a C_q_ (quantification cycle) value standard deviation (*SD*) higher than 1 was considered as inconsistent (Pfaffl, Tichopád, Prgomet, & Neuvians, 2004). Thus, *arp1* (*SD*: 1.37), *rpS5α* (*SD*: 1.27), *rp49* (*SD*: 1.16), and *ppil2* (*SD*: 1.28) had to be rejected as reference genes. Consequently, only *pros26* (*SD*: 0.77) was satisfactory as reference gene from the initial testing group. In addition, *mrjp8* (*SD*: 0.97) showed a very stable expression within the analyzed samples and was used as second reference gene whereas all other target genes (*mrjp1‐7*, *mrjp9,* and *apisimin*) were regulated over a wide range (*SD*: 1.95–5.47).

C_q_ values were determined with the Bio‐Rad CFX Manager 3.1 (Bio‐Rad) using linear regression for each sample with C_q_ determination mode. Specificity of qPCR products was analyzed with the capillary electrophoresis system QIAxcel (Qiagen) (Figure S2). PCR efficiency was estimated by serial dilution qPCR (Table S1), and relative target gene expression was determined according to Pfaffl (2001) using the geometric mean of the reference genes *mrjp8* and *pros26*. To determine relative transcript abundance (Table S2), relative gene expression was normalized to total RNA amount by multiplication of both values.

### 2.3 Protein isolation and SDS–polyacrylamide (PA) gel electrophoresis (GE)

Proteins were precipitated from the flow‐through after binding of the RNA to the NucleoSpin^®^ RNA columns (NucleoSpin^®^ RNA Kit, Macherey‐Nagel) by sodium deoxycholate/trichloroacetic acid according to Arnold and Ulbrich‐Hofmann (1999). Of the ten samples isolated per tissue on days zero and eight, five were retained for subsequent quantitative mass spectrometry (see Section 2.4). For SDS‐PAGE, individual protein pellets were dissolved in 15 µl sample buffer (100 mM Tris/HCl, 4.8% (*w*/*v*) SDS, 16% (*v*/*v*) glycerol, 0.1% (*w*/*v*) bromophenol blue, 2% (*v*/*v*) β‐mercaptoethanol, pH 8.0) and analyzed in 8% acrylamide gels (Laemmli, 1970) at 175 V for 60 min. RJ protein extract used as reference was prepared from frozen RJ (Naturprodukte Lembcke GbR) according to Buttstedt, Ihling, Pietzsch, and Moritz (2016). Unstained Protein Marker, Broad Range (10–200 kDa) (New England Biolabs) was used as protein marker, and gels were stained with Coomassie Brilliant Blue G250 (Neuhoff, Arold, Taube, & Ehrhardt, 1988). Protein bands cut from SDS‐PA gels were identified via mass spectrometry (ESI‐QTOF‐MS/MS) according to Pamminger et al. (2016).

### 2.4 Quantitative mass spectrometric analyses

Nano‐LC‐HD‐MSE data were finally acquired for three randomly selected biological replicates per group (HGs and brains, day 0 and day 8) and three technical replicates for each biological replicate. Quantitative mass spectrometric analyses were performed through principles described earlier (Helm, Dobritzsch, Rödiger, Agne, & Baginsky, 2014). Briefly, 1 ul of tryptic peptides (~400 ng peptides) was trapped on a 20 mm × 180 um fused silica M‐Class C18 trap column (Waters) and washed for 5 min at 5 µl/min with a solution of 1% acetonitrile (ACN, containing 0.1% formic acid [FA]) in 99% water (containing 0.1% trifluoroacetic acid). Afterwards, the peptides were separated on a 250 mm × 75 um fused silica M‐Class HSS T3 C18 column (with 1.8 um particle size) (Waters) over a 120‐min gradient consisting of increasing concentrations of 7%–40% of 0.1% FA in ACN within 0.1% FA in water (Carl Roth). Eluting peptides were ionized at 2.1 kV from a precut PicoTip Emitter (New Objective) with source settings of 80 C and nano N2 flow of 0.4 bar. Ions passed into the SYNAPT G2‐S Mass Spectrometer (Waters) which was operated in both positive ion mode and resolution mode, and with the following settings: ion trap cell mobility separation with a release time of 500 μs, and afterward “cooled” for 1,000 μs; helium pressure set to 4.7 mbar and IMS cell nitrogen pressure to 2.87 mbar; wave height was 38 V; and wave velocity ramped from 1,200 to 400 m/s. Glu‐1‐fibrinopeptide B (250 fmol/μL, 0.3 μL/min) was used as lock mass (m/z = 785.8426, z = 2).

Data analysis was carried out by ProteinLynx Global SERVER (PLGS 3.0.1, Waters) with automated determination of chromatographic peak width and MS TOF resolution. Lock mass value for charge state two was 785.8426 Da/e with lock mass window of 0.25 Da, low/high energy threshold of 250/100 counts, and intensity threshold of 750 counts. The characteristics of the peptide and protein matching were set to be 2 for minimal number of fragment ion matches for each peptide match, 5 for minimal number of fragment matches to a protein, and 2 as the minimal number of matched peptides per identified protein. The detection limit of the method was quantified as 0.023 fmol using phosphorylase B. The most abundant protein in this study is MRJP1 on day 8 in the HGs with an average quantity of 66.4 fmol, and the least abundant protein is phenoloxidase subunit A with 0.043 fmol. These values are in very good accordance with those published by Helm et al. (2014).

During mass spectrometric analysis, 1,364 of the 1,552 proteins/protein isoforms in the database (see Supplementary Material and Methods section) (Table S3) were quantified in at least one of the twelve samples analyzed (2 days (0 and 8), 2 tissues (HGs and brains), 3 replicates each) (Table S4, Tab “All detected proteins”). If a protein in the database was not quantified in a sample, the amount was set to zero for statistical analyses. It should be noted that this does not necessarily mean that the protein was not present in the sample; its amount might be just too low for quantification. For statistical analyses, the final protein list (Table S4) was further adapted as described in the Supplementary Material and Methods section. The final database included 1,552 different proteins/protein isoforms (Table S3, Tab “New database for mass spec”). All mass spectrometry data have been deposited to the ProteomeXchange Consortium (http://proteomecentral.proteomexchange.org) via the PRIDE partner repository (Vizcaino et al., 2013) with the dataset identifier PXD012618.

### 2.5 Statistics

All statistical analyses were performed with Statistica 8.0 (StatSoft). Total RNA amount data were log‐transformed to achieve normal distribution (Kolmogorov–Smirnov test, *p* > 0.05) and subsequently analyzed via a full factorial analysis of variance (ANOVA) with post hoc Bonferroni test. Spearman's rank correlations (*ρ*) were performed between the different genes using relative gene expression data and between relative transcript abundance and protein amount. In the latter case, we correlate mRNA with proteins, and thus, an unusual spelling italic and capital letters is used to name *gene* and PROTEIN; for example, *MRJP1* refers to correlation of *mrjp1* mRNA transcript abundance with MRJP1 protein amount. For the statistical analyses of relative transcript abundances, values were Box‐Cox‐transformed to meet criteria of normal distribution (Kolmogorov–Smirnov test, *p* > 0.05) and a general linear model (GLM) was performed to reveal major effects (gene, age, tissue, and interactions between these effects). Within gene, comparisons over time were analyzed via one‐way ANOVAs with post hoc Bonferroni tests. To show the extent of variability in relation to the mean, coefficients of variation were calculated for the relative transcript abundances by dividing the standard deviation (*SD*) with the mean. This allows for comparing the variation of samples despite different means. A coefficient of variation of 1.0 indicates that the *SD* is 100% of the mean, for example, 1.0 ± 1.0.

Mass spectrometry data did not meet criteria for normal distribution and were thus analyzed with a generalized linear model (GZLM) to reveal major effects (protein, age, tissue, and interactions between these effects). A Venn diagram was generated using VENNY 2.1 to illustrate tissue and time dependent changes (Oliveros, 2007). Heat maps were built using the MultiExperiment Viewer (MeV, mev.tm4.org) version 4.9, and hierarchical clustering was performed using optimized gene and sample leaf order, Euclidean distance, and average linkage clustering (bootstrap resampling, 1,000 replications) (Eisen, Spellman, Brown, & Botstein, 1998).

## 3 RESULTS

### 3.1 Gene expression

**Figure 2:**
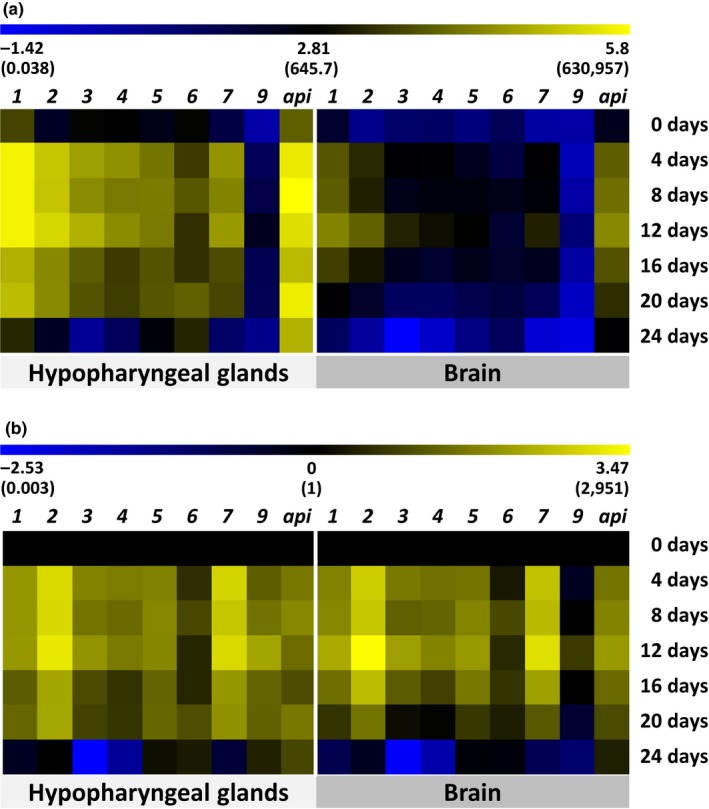
Heat map of relative transcript abundances in the hypopharyngeal glands and brains of worker honeybees. Transcript abundance is represented for all genes as a color gradient across all samples from light yellow (highest) to deep blue (lowest). Values were log‐transformed (nontransformed in brackets) and visualized using the MultiExperiment Viewer (MeV, mev.tm4.org) version 4.9. *Mrjp8* is missing in the transcriptional analysis as it was so evenly expressed and neither influenced by tissue nor by age of the bees that *mrjp8* was used as second reference gene in addition to *pros26*. (a) Relative transcript abundance from day 0 to day 24 in the hypopharyngeal glands and the brains. (b) Relative transcript abundance normalized to freshly hatched worker bees (0 d). (*1‐9*, *mrjp1‐9*; *api*, *apisimin*; d, days; *n* = 3 pools of 3 bees per day and tissue)

Figure 2 shows the relative transcript abundances of *mrjp1‐7*, *mrjp9,* and *apisimin* which were influenced by a number of factors including gene, age of the bees, and tissue (GLM; gene: *F* = 234.75, *df* = 8, *p* < 0.001; age: *F* = 258.84, *df* = 6,* p* < 0.001; tissue: *F* = 1,427.57, *df* = 1, *p* < 0.001). Interactions were found between tissue and gene (GLM; *F* = 3.71, *df* = 8, *p* < 0.001) and tissue and age (GLM; *F* = 4.94, *df* = 6, *p* < 0.001) which is attributed to the fact that relative transcript abundance was, except for *mrjp9* on day 0, for all genes and all days higher in the HGs than in the brains (9.8‐ (*apisimin* on day 12) to 357.9‐fold (*mrjp3* on day 20), Figure 2a). A further interaction was found between gene and age (GLM; *F* = 8.96, *df* = 48, *p* < 0.001) as most genes were differentially regulated over time.

In the HGs, relative transcript abundance for all examined genes increased from day 0 to days 4–12 and decreased again until day 24 (one‐way ANOVA, *p* < 0.001, *df* = 125, *F* = 18.46) (Figure 2b, Table 1). Furthermore, day 0 and day 24 did not differ significantly in transcript abundances for any of the genes (Table 1). During the nurse bee period (days 4–12), transcript abundances between the genes differed remarkably with *mrjp1* (483,887 ± 120,413 (mean of relative transcript abundance at days 4–12 ± *SD*)) and *apisimin* (425,101 ± 208,541) being highest expressed, followed by *mrjp2* (166,575 ± 69,058), *mrjp3* (51,511 ± 29,132), *mrjp7* (34,258 ± 14,151), *mrjp4* (26,780 ± 9,588), *mrjp5* (17,323 ± 4,903), *mrjp6* (4,259 ± 2,217), and *mrjp9* (88 ± 92) (Table S2). Except for *mrjp6*, all the genes had their highest transcript abundances within one of these nurse bee days (Table 1). The exceptional case *mrjp6* showed highest transcript abundance at day 20 (Table 1) accompanied by a lower transcript increase from the day of hatching to days 4, 8, and 12 (~3‐ to 10‐fold) compared to any other gene (~20‐ to 1,500‐fold).

**Table 1:** Relative transcript abundance of a specific gene over all analyzed days within tissue

	Relative *mrjp* transcript abundance within tissues													
	Hypopharyngeal glands							Brains						
*mrjp1*	0^b^	**4^a^**	**8^a^**	**12^a^**	16^b^	20^b^	24^b^	0^c,d^	**4^a,b^**	**8^a^**	**12^a^**	**16^a,b,c^**	20^b,c,d^	24^d^
*mrjp2*	0^d^	**4^a,b^**	**8^a,b,c^**	**12^a^**	16^b,c,d^	20^c,d^	24^d^	0^c^	**4^a^**	**8^a,b^**	**12^a^**	**16^a,b^**	20^b,c^	24^c^
*mrjp3*	0^b,c^	**4^a^**	**8^a,b^**	**12^a^**	**16^a,b,c^**	20^b,c^	24^c^	0^b,c,d^	**4^a,b^**	**8^a,b,c^**	**12^a^**	**16^a,b,c^**	20^c,d^	24^d^
*mrjp4*	0^c,d^	**4^a^**	**8^a,b^**	**12^a^**	16^b,c^	20^c,d^	24^d^	0^c,d^	**4^a,b^**	**8^a,b^**	**12^a^**	16^b,c^	20^c,d^	24^d^
*mrjp5*	0^b^	**4^a^**	**8^a^**	**12^a^**	**16^a,b^**	**20^a,b^**	24^b^	0^c^	**4^a,b^**	**8^a^**	**12^a^**	**16^a,b^**	20^b,c^	24^c^
*mrjp6*	0^c^	4^b^	**8^a^**	12^b,c^	16^b,c^	**20^a^**	24^b,c^	**0**	**4**	**8**	**12**	**16**	**20**	**24**
*mrjp7*	0^c,d^	**4^a^**	**8^a,b^**	**12^a^**	16^b^	20^b,c^	24^d^	0^d^	4^a,b^	8^a,b^	**12^a^**	16^b,c^	20^c,d^	24^d^
*mrjp9*	0^d^	4^b,c^	**8^a,b^**	**12^a^**	16^b^	20^b,c^	24^c,d^	**0^a,b^**	4^b,c^	**8^a,b^**	**12^a^**	**16^a,b^**	20^b,c^	24^c^
*apisimin*	0^c^	**4^a,b^**	**8^a^**	**12^a,b,c^**	16^b,c^	**20^a,b^**	24^b,c^	0^d^	**4^a,b^**	**8^a,b^**	**12^a^**	**16^a,b,c^**	20^b,c,d^	24^c,d^

Note

Box‐Cox‐transformed data were analyzed with one‐way ANOVA followed by post hoc Bonferroni test. ^a‐d^Transcript abundances of days in the same row with different superscripts are significantly different (*p* < 0.05). The day with the highest transcript abundance for a specific gene is highlighted in gray, and days that do not differ from the day with the highest transcript abundance are depicted in bold.

In the brain, expression of *mrjp1‐5*, *mrjp7,* and *apisimin* followed the same pattern as already observed in the HGs with a relative transcript abundance increase from days 0 to 12 and a further decrease to day 24 (Figure 2b, Table 1, Table S2). All of these genes were highest expressed at day 12 (Table 1) again showing remarkable differences in transcript abundance between the genes but following the same order as in the HGs (*apisimin—*27,093 ± 26,073; *mrjp1—*23,302 ± 19,178; *mrjp2—*9,108 ± 7,178; *mrjp3—*1,696 ± 848; *mrjp7—*1,589 ± 1,020; *mrjp4—*944 ± 554; *mrjp5—*689 ± 360; *mrjp6—*87 ± 56; and *mrjp9—*8 ± 4). Albeit highest expressed at day 12, transcript abundance of *mrjp9* did not significantly increase from days 0 to 12 but showed a decrease at day 24 (Figure 2b, Table 1). *Mrjp6* did not show any significant difference in transcript abundance in the brain.

Because of the known complex formation of MRJP1 and apisimin in RJ with a stoichiometry of 4:4 (Mandacaru et al., 2017; Tian et al., 2018), we explicitly compared transcript abundance of these two genes which did not differ within the same day between days 0 and 20, but *apisimin* had significantly more transcripts than *mrjp1* on day 24 in both brains and HGs (HG: 44‐fold (*p* < 0.001); brain: 32‐fold (*p* = 0.034)) (one‐way ANOVA, *p* < 0.001, *df* = 27, *F* = 43.046; see Figure S3).

The expression of all the genes, except for *mrjp3* with *mrjp6*, correlated significantly (Table S5). In general, very high correlations (Spearman's ρ ≥ 0.90) were observed between *mrjp1‐4* and *mrjp7* (Table S5). Within the group of *mrjp1‐7*, *mrjp6* was the only one with correlation factors below 0.7 (0.37 < *ρ* < 0.66).

When calculating the relative transcript abundances, conspicuous high differences between some of the pools of the same day and gene attracted our attention. As a measure of relative variability, the coefficient of variation was calculated (Table 2). In the HGs, high (≥0.8, highlighted in bold) and very high (≥1.0, highlighted in italics) coefficients of variation were observed for *mrjp1‐4* and *mrjp7* on day 20, *mrjp2*‐*4* and *mrjp7* on days 16 and 24, and *mrjp9* on day 9. In the brain, on day 20 *mrjp1‐4* and *mrjp7*, as well as on day 24 *mrjp2*, *mrjp4,* and *mrjp7*, and on day 8 *mrjp6* showed very high coefficients of variation (≥1.0, highlighted in bold) (Table 2).

**Table 2:** Coefficients of variation (ratio of standard deviation to mean) for *mrjp1‐7*, *mrjp9,* and *apisimin* transcript abundances

Tissue	Hypopharyngeal glands							Brains						
Day	0	4	8	12	16	20	24	0	4	8	12	16	20	24
*mrjp1*	0.49	0.06	0.44	0.23	0.72	**1.13**	0.69	0.63	0.53	0.57	*0.82*	0.76	**1.07**	*0.97*
*mrjp2*	0.65	0.15	0.53	0.37	*0.81*	**1.32**	**1.30**	0.74	0.72	0.36	0.79	0.78	**1.36**	**1.32**
*mrjp3*	0.50	0.26	0.46	0.43	*0.96*	**1.65**	**1.42**	*0.85*	*0.89*	0.50	0.50	*0.86*	**1.64**	*0.99*
*mrjp4*	0.34	0.26	0.37	0.28	*0.80*	**1.39**	**1.12**	0.56	0.62	0.46	0.59	0.79	**1.38**	**1.05**
*mrjp5*	0.29	0.40	0.29	0.25	0.40	0.71	0.68	0.54	0.57	0.74	0.52	0.59	0.39	*0.91*
*mrjp6*	0.34	0.11	0.16	0.17	0.29	0.15	0.14	*0.83*	0.38	**1.19**	0.64	0.28	0.42	0.77
*mrjp7*	0.47	0.32	0.53	0.34	*0.82*	**1.27**	**1.54**	0.63	0.66	0.43	0.64	*0.81*	**1.17**	**1.22**
*mrjp9*	0.22	*0.88*	0.61	0.30	0.48	0.76	0.52	*0.81*	0.56	0.67	0.58	0.66	0.61	0.43
*apisimin*	0.34	0.37	0.34	0.18	0.23	0.68	0.30	0.49	0.29	0.66	*0.96*	0.41	0.19	0.59

Note

High (≥0.80) and very high (≥1.00) coefficients of variation are indicated in bold and italics, respectively.

### 3.2 Protein amounts

The protein amounts isolated per single brain or per pair of HGs were more than sufficient to be analyzed by quantitative mass spectrometry (Figure S4). Within the HGs, the high abundance of MRJP1 at day 8 is already visible on SDS‐PA gels. Interestingly, the band corresponding to MRJP1, directly isolated from the HGs, migrates at a lower apparent molecular weight (~50 kDa) than when isolated from RJ (~55 kDa) (Figure S4, MRJP1 marked with asterisk). This discrepancy was already observed upon the first isolation of MRJP1 (Hanes & Šimúth, 1992) but has not yet been clarified. The two other conspicuous bands (~40 and 200 kDa), primarily found in the brains (Figure S4), were identified as myosin heavy chain (~200 kDa, Gene ID: 409843) and actin‐related protein 1 (~40 kDa, Gene ID: 406122).

In total, 1,003 proteins were quantified (Table S4, Figure S5). In the HGs, less proteins were quantified at day 0 (253) than at day 8 (679) whereas in the brain the number of quantified proteins was almost equal on both days (767*—*brain at day 0; 758*—*brain at day 8). Regarding tissue specificity, 309 proteins were brain‐specific but only 114 were HG‐specific. Furthermore, 62 proteins were only quantified at day 0 whereas 218 proteins were specifically found at day 8 (Table S4, Figure S5). The dendrogram (Figure S6) based on the amounts of all detected proteins reveals two distinct clusters, one comprising both brain samples (days 0 and 8) and one comprising both HG samples (days 0 and 8). Thus, the samples cluster according to tissue and not according to age of the worker bees. The hierarchical protein‐wise clustering illustrates that MRJP1 and MRJP3 can be found in main cluster 1, separated from all other quantified MRJPs in main cluster 4 (Figure S6). More results on proteins other than MRJPs can be found in the Supplementary Results section.

**Figure 3:**
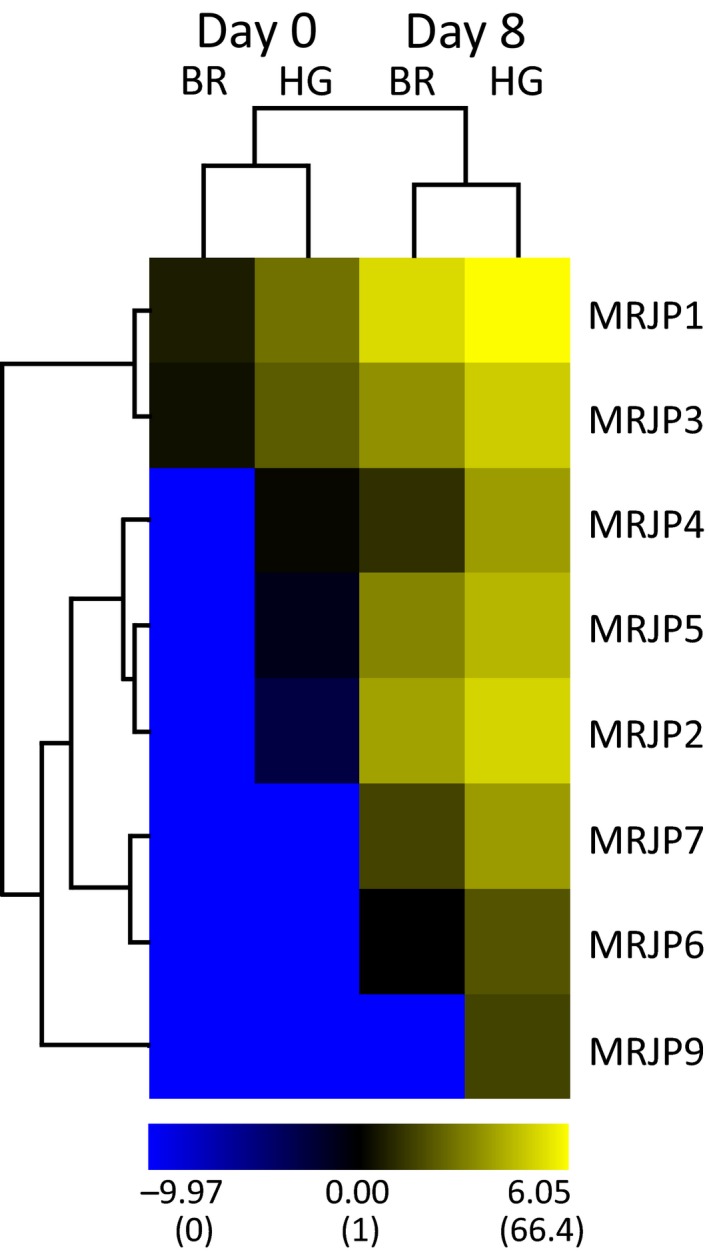
Heat map of mean protein amounts (fmol on column) in the hypopharyngeal glands (HG) and brains (BR) of worker honeybees at day zero and eight. Protein amounts are represented as a color gradient from light yellow (highest) to deep blue (lowest; here: not detected). Values were log2‐transformed (nontransformed in brackets) and visualized using the MultiExperiment Viewer (MeV, mev.tm4.org) version 4.9 (*n* = 3 per day and tissue)

Of the nine MRJPs, MRJP8 was the only one that was not quantified within a single sample. For all other MRJPs, protein amounts were strongly affected by protein type, age of the bees, and tissue (GZLM; protein: W = 197.29, *df* = 7, *p* < 0.001; age: W = 333.98, *df* = 1,* p* < 0.001; tissue: W = 46.45, *df* = 1, *p* < 0.001). In addition, an interaction was found between age and protein (W = 41.36, *df* = 7, *p* < 0.001) as all MRJPs either were found in higher amounts at day 8 compared to day 0 (6.5‐ to 209.6‐fold) or were only detected at day 8 but not at day 0 (Figure 3). An interaction was also found between age and tissue based on the independent increase in protein amounts from days 0 to 8 (Figure S4) in both tissues (W = 5.83, *df* = 1, *p* = 0.016). This general increase in MRJP amount at day 8 leads, in contrast to the samples containing all quantified proteins (Figure S6), to clustering according to age of bees and not according to tissue (Figure 3). As already observed within the hierarchical clustering of all quantified proteins (Figure S6), MRJP1 and MRJP3 build a separate cluster apart from all other MRJPs (Figure 3).

As MRJP1 and apisimin form in RJ a complex with a stoichiometry of 4:4 (Mandacaru et al., 2017; Tian et al., 2018) and as relative transcript abundance is similar for both genes (Figure S3), one would expect to quantify approximately the same molar amount of apisimin as MRJP1 at least in the HG at day 8. However, within the present study apisimin was not detected at all.

Due to generally low values at day 0 and higher values at day 8, overall transcript abundance correlated significantly with quantified protein amounts (Spearman's *ρ* = 0.739, *p* < 0.001). Gene‐wise comparisons revealed high correlations (*ρ* > 0.8, *p* < 0.001) for *MRJP1* (*ρ* = 0.872, *p* < 0.001), *MRJP2* (*ρ* = 0.863, *p* < 0.001), and *MRJP7* (*ρ* = 0.832, *p* < 0.001) and medium correlations (0.6 < ρ < 0.8, 0.01 < *p* < 0.05) for *MRJP3* (ρ = 0.601, *p* = 0.039), *MRJP4* (*ρ* = 0.696, *p* = 0.012), *MRJP5* (*ρ* = 0.689, *p* = 0.013), and *MRJP9* (*ρ* = 0.640, *p* = 0.025). For *MRJP6*, transcript abundance did not significantly correlate with protein amount (*ρ* = 0.408, *p* = 0.188).

At day 8, MRJPs represent a total of 185.2 ± 81.8 fmol protein (mean of fmol sum of all MRJPs ± *SD*) and thus 8.4% of all proteins in the HGs. MRJP1 is the most abundant protein (66.4 ± 5.8 fmol), but also MRJP2 (34.3 ± 22.8 fmol; 3rd place), MRJP3 (29.7 ± 24.4 fmol; 5th place), and MRJP5 (20.9 ± 10.2 fmol; 13th place) were found within the 20 most abundant proteins (Table S6). Within the brain, only MRJP1 was found within the 20 most abundant proteins at day 8 (37.8 ± 9.8 fmol; 5th place) (Table S6).

## 4 DISCUSSION

We here studied, along the time gradient of individual worker bee development, the potential involvement of MRJPs in caste‐specific phenotypic plasticity in combination with developmental variance. Whereas the expression of some *mrjps* changed with age, others do not seem to be influenced by age or age polyethism:

*Mrjp8* showed a very low and even expression (C_q_ = 26.75 ± 0.97, mean ± *SD*), and the protein could not be quantified neither in the brain nor in the HGs. Among the other *mrjps*, *mrjp9* is the lowest expressed *mrjp* at any time point in both tissues (Figure 2a, Table S2) and only low amounts of the protein (3.1 ± 2.9 fmol) were quantified solely at day 8 in the HGs. Concordant with that, MRJP8 and MRJP9 were not detected in a comparative proteome study of nurse and forager bee brains (Hernández et al., 2012). Albeit detectable in royal jelly (Zhang et al., 2014), MRJP8 and MRJP9 only represent a minor portion as MRJP1‐3 and MRJP5 account for 82%–90% of total food jelly proteins (Schmitzová et al., 1998). Taken together with the fact that *mrjp8* and *mrjp9* were defined as the most ancestral *mrjp* gene pair (Buttstedt et al., 2013, 2014; Helbing et al., 2017) and that they were also identified as components of honeybee venom (Peiren et al., 2005, 2008), a tissue‐specific function in the HGs or the brain and thus an involvement in phenotypic plasticity seem unlikely.

*Mrjp1‐7* show in the HGs an age‐dependent expression pattern with an increase in transcript abundance from day zero to days 4–12 and a subsequent decrease to day 24 (Figure 2, Table 1). This is in accord with the general notion that *mrjps* are higher expressed in brood‐feeding nurse bees than in foragers (Klaudiny et al., 1994; Kubo et al., 1996; Ohashi et al., 1997). However, whereas all other *mrjps* do have their highest transcript abundance during the nurse bee period, *mrjp6* shows highest abundance at day 20 (Table 1) in accordance with previous studies reporting on a significantly higher expression of *mrjp6* in forager compared to nurse bee heads and HGs (Buttstedt et al., 2013; Liu et al., 2014). In the brain, *mrjp6* expression does not show any caste‐related modulation supported by Hernández et al. (2012) who did not detect differences in MRJP6 between brains of nurses and foragers. *Mrjp6* differs in its time‐resolved expression pattern clearly from the other head‐expressed *mrjp1‐5* and *mrjp7,* and thus, a coregulation of all head‐expressed *mrjps* by the very same transcription factors is unlikely. This is supported by Winkler et al. (2018) who were able to downregulate the expression of *mrjp1‐3* with 20‐hydroxyecdysone, a molting hormone in insects, whereas *mrjp4*‐*9* were not affected (Winkler et al., 2018). However, *mrjp6* expression in heads of foragers and nurses is higher than in heads of drones and queens (Buttstedt et al., 2013). Thus, although a nurse‐specific function is unlikely, the focus of expression lays in the heads of workers whereas the reproductive castes express 200‐ to 6,500‐fold less *mrjp6* in their heads (Buttstedt et al., 2013).

At day 8, MRJPs represent 8.4% of all proteins in the HGs. In royal jelly, MRJPs account for 82%–90% of all proteins (Schmitzová et al., 1998). This difference is due to the fact that royal jelly only contains the proteins that are secreted by the HGs, whereas in our study whole HGs were used as sample. As expected, at day eight MRJP1‐3 (66.4–29.7 fmol) and MRJP5 (20.9 fmol) are among the 20 most abundant HG proteins (Table S6). Interestingly, also MRJP4 and MRJP7 were found in medium quantities (13.5 and 13.3 fmol, respectively) albeit both proteins were not detected in early proteome studies on royal jelly (Li, Feng, Zhang, & Pan, 2008; Scarselli et al., 2005; Schmitzová et al., 1998). However, studies that are more recent confirm the presence of MRJP4 and MRJP7 in royal jelly (Feng et al., 2015; Zhang et al., 2014). MRJP1‐7 were also quantified in the honeybee brain at day eight, and albeit detected in lesser amounts than in the HGs (1.8‐fold), MRJP1 is the fifth most abundant protein at day eight in the brain. Its amount increased from hatching to the nurse bee period 23.4‐fold (Table S4) and has been shown to decrease again 9.2‐fold from the nurse bee to the forager state (Garcia et al., 2009). Within the brain, immunolocalization revealed MRJP1 to be present in the antennal and the optical lobes, and in the intercellular spaces in mushroom bodies (Garcia et al., 2009; Kucharski, Maleszka, Hayward, & Ball, 1998; Meng et al., 2018), brain structures involved in associative learning (Menzel, 1993). In the buff‐tailed bumblebee *Bombus terrestris,* the single‐copy *mrjp‐like* (*mrjpl*) gene is also transcribed in the brain (NCBI database BioProject PRJNA383917) and the protein can be immunohistochemically detected in the mushroom bodies (Albert, Spaethe, Grübel, & Rössler, 2014). Albert et al. (2014) suggest therefore that expression of *mrjpls* in the brain corresponds to the ancestral function rather than to a derived one. However, in *A. mellifera* the ancestral *mrjp9* is not upregulated in heads compared to thoraces and abdomen of workers (Buttstedt et al., 2013), and based on our data, we excluded a tissue‐specific function of the ancestral MRJP9 in the brain. However, this does not exclude that the single‐copy ancestral *mrjpl* indeed fulfills, among others, a brain‐specific function. This function might have been lost in the honeybee, as new *mrjp* copies, for example, MRJP1, adopted these functions. MRJP1 can regulate and affect the growth of cells across species (Wan et al., 2018; Watanabe et al., 1996), and thus, the protein might be involved in growth regulation of specific neurons (Kenyon cells) in the mushroom bodies, an idea already raised by Albert et al. (2014).

In royal jelly, the crucial viscosity‐determining function of MRJP1 (Buttstedt et al., 2018) is only accomplished by oligomer and subsequent fibril formation of MRJP1 together with apisimin (Buttstedt et al., 2018; Mandacaru et al., 2017). The gene encoding *apisimin* has up to date only been found in the genus *Apis* and thus seems to be an orphan within the genus with the only so far described function in complex with MRJP1. Both genes show high and similar expression values until day 20 not only in the HGs but also in the brain (Figures 2, S3). However, we were not able to quantify the apisimin protein via mass spectrometry neither in the HGs nor in the brain. This is due to the fact that the 54 amino acids comprising apisimin possess only two unfavorably situated trypsin cleavage sites, resulting after trypsin cleavage in a single lysine, a hexapeptide of only 633.74 Da, and the residual 47 amino acids of the protein (4,796.49 Da). Thus, identification, for which at least two peptides are needed, and subsequent quantification were not possible with a tryptic digest. Furthermore, the plain identification of apisimin via mass spectrometry has been described to be difficult before (Buttstedt et al., 2018; Tamura et al., 2009).

*Mrjp1‐5* and *mrjp7* show not only in the HGs but also in the brain an expression increase from hatching until the nurse bee period and then a subsequent decrease to day 24. Thus, the initial question, whether the observed elevated expression of *mrjp1*, *mrjp2*, *mrjp5,* and *mrjp7* in forager heads is caused by a shift of expression from the HGs to the brain while the bees age, can be clearly answered with no. Indeed, for *MRJP5* all scenarios were described: significantly higher in foragers than nurses (Hernández et al., 2012 (brain)), no difference between nurses and foragers (Buttstedt et al., 2013 (heads); Ji et al., 2014 (HGs)), or being higher in nurses than in foragers (Drapeau et al., 2006 (heads)). *MRJP1*‐*4* and *MRJP7* are in the majority of studies described as being higher in nurse bees than in foragers with some studies detecting the proteins exclusively in nurses (Feng, Fang, & Li, 2009 (HG); Garcia et al., 2009 (brain); Hernández et al., 2012 (brain); Hu et al., 2019 (HG); Ji et al., 2014 (HG) Klaudiny et al., 1994 (head); Kubo et al., 1996 (HG); Liu et al., 2013 (HG); Ohashi et al., 1997 (HG); Peixoto et al., 2009 (brain)).

But why are there these discrepancies between studies? Indeed, also in Buttstedt et al. (2013) expression of *mrjp1*, *mrjp2,* and *mrjp7* was found to be fourfold to 36‐fold lower in forager compared to nurse bee heads; however, these differences were deemed not significant due to high standard errors, particularly in forager heads (Buttstedt et al., 2013). Also, in this study, very high (≥1.0) coefficients of variation were, except for *mrjp6* on day 8 in the brain, exclusively observed for *mrjp1‐4* and *mrjp7* within the forager period at days 20 and 24 (Table 2). In addition, on day 16 in the HGs coefficients of variations for *mrjp2‐4* and *mrjp7* are high (≥0.8). This indicates that individual variation especially for *mrjp1‐4* and *mrjp7* is high during the transition phase from nursing to foraging. The cause for this is that the transition is not a sudden shift but rather characterized by a transition phase where nursing slowly fades out and foraging gradually begins (Seeley, 1982). The physiological changes that workers undergo during age‐related polyethism seem to be uniquely timed for each individual worker. Indeed, although the majority of bees start to forage at an age around 20 days, individual bees are known to fly out for their first collecting flights as early as day 10 (Rösch, 1925; Seeley, 1982; zu Oettingen‐Spielberg, 1949). In addition, whereas some bees do not continue to feed brood after starting to forage, others do so (Rösch, 1925). Thus, during the transition phase feeding larvae and foraging are not mutually exclusive. So it is not possible to be sure that a specific bee is investing most of its time foraging instead of raising larvae either when collecting age marked bees or when collecting foragers identified by carrying pollen or propolis on their hind legs. For example, the highest coefficient of variance (1.65) was measured for *mrjp3* on day 20 in the HGs. Here, relative transcript abundances of the individual pools are 7.3, 582, and 18,415. A transcript abundance of 7.3 is similar to the transcript abundances measured on day 24 (0.4–5.8), whereas 18,415 lies within the range of the transcript abundances detected at day 8 directly within the nurse bee period (14,221–39,649). This explains discrepancies that, for example, MRJP1 is described as “detected in the nurse‐bee gland, but not in the forager‐bee gland” (Kubo et al., 1996) or as “detected in both the nurse‐bee and forager‐bee glands, although its density was stronger in the nurse‐bee gland” (Ohashi et al., 1997). Thus, conclusions on expression of *mrjps* should be made with caution, especially when working with forager bees.

Taken together, our results in combination with previous studies suggest the following: Time‐resolved expression in the brain follows for each *mrjp* the expression in the HGs, that is, when expression increases in the HGs, an expression increase is also seen in the brain. However, expression in the brain is always lower than in the HGs (19.4‐ (*mrjp6* on day 16) to 357.9‐fold (*mrjp3* on day 20), Figure 2a). In both tissues, the *mrjps* can be divided into several groups: (I) *Mrjp1‐4* and *mrjp7* show after hatching of the bees an upregulation throughout the nurse bee period and a further downregulation until the forager state. However, individual variance is high, especially during the transition phase from nursing to foraging. (II) *Mrjp5* follows in this study the scenario mentioned afore but without showing high (≥0.80) coefficients of variation. However, in previous studies it was described as higher in nurses than foragers, being similar between nurses and foragers, and being higher in foragers than in nurses (Buttstedt et al., 2013; Drapeau et al., 2006; Hernández et al., 2012; Ji et al., 2014). The cause of these differences is still unclear. (III) *Mrjp6* expression does not vary significantly in the brain and shows its highest expression in the HGs during the forager bee period. (IV) *Mrjp8* and *mrjp9* show, compared to the other *mrjps*, low expression in the HGs and in the brain. Due to their higher occurrence in other body parts of the bees (Buttstedt et al., 2013; Peiren et al., 2005, 2008), they might not have a HG‐ or brain‐specific function.

Thus, whereas an involvement of *mrjp1‐7* in caste‐specific phenotypic plasticity is possible, especially *mrjp8* and *mrjp9* do not seem to be influenced by age polyethism.

## Conflict of interest

None decalred.

## **AUTHOR** **CONTRIBUTIONS**

DD and AB designed the research; DD, DA, and AB performed the research; DD, SE, MF, and AB analyzed the data; AB prepared the original draft; and DD, DA, SE, and AB reviewed and edited the first manuscript version. All authors reviewed and edited the revised version.

## Data Availability

The mass spectrometry proteomics data have been deposited to the ProteomeXchange Consortium (http://proteomecentral.proteomexchange.org) via the PRIDE partner repository (Vizcaino et al., 2013) with the dataset identifier PXD012618. Transcriptomic data are provided in the Table S2.
