# Supplementary material for: The rise and fall of major royal jelly proteins during a honeybee (Apis mellifera) workers' life
Source: Ecol Evol. 2019 Jul 5;9(15):8771–82. doi: 10.1002/ece3.5429 (PMC6686338; doi:10.1002/ece3.5429)
Supplement: Supplementary file 3 [file ECE3-9-8771-s003.pdf]

Supporting information

for

The rise and fall of major royal jelly proteins during a honey bee (*Apis mellifera*) workers' life

Dirk Dobritzsch, Denise Aumer, Matthew Fuszard, Silvio Erler & Anja Buttstedt

1. Supplementary material and methods..2

2. Supplementary results...3

3. Supplementary discussion...4

4. Supplementary figures and tables 6

Figure S1: Total RNA isolated per HG pair or per brain...6

Figure S2: Analysis of qPCR product specificity...7

Figure S3: Relative transcript abundance of *apisimin* and *mrjp1*.......................................7

Figure S4: SDS PA gels showing the proteins isolated per pair of HGs and per brain........8

Figure S5: Venn diagram of proteins within the HGs and the brain....................................9

Figure S6: Heat map of mean protein amounts in the HGs and brain...............................10

Table S1: Primers used for qPCR and PCR efficiency..11

Table S2: Relative transcript abundances for *mrjp1-7, 9* and *apisimin*.............................12

Table S3: Database for mass spectrometric analyses (separate excel file).

Table S4: Results of quantitative mass spectrometry (separate excel file).

Table S5: Correlation matrix for *mrjp1-7*, *9* and *apisimin*……………………………….13

Table S6: Protein amounts of the 20 most abundant proteins...14

4. Additional supplementary references...15

1. Supplementary material and methods

Adaptation of the final protein list

1) Proteins quantified with a yellow (maybe) or red (poor) auto-curate threshold by PLGS (where several factors contribute search scores, such as number of entries in the database, comparison of peptide or fragment masses calculated from database with the measured data, peak area, number of matched vs. unmatched peptides, fragmentation characteristics of peptides, encoded into a Markov model that incorporates a, b, y and immonium ions, fragment ions, from modifications, and internal ions from proline, as well as search parameters including proteases, number of missed cleavages, fixed and variable modifications.) are usually considered of insufficient quality and are excluded from further analysis (requirements for "OK" assignment: 95% probability, for "maybe" 50%). For our data, within the twelve values per protein in many cases at least one was identified with a yellow or red auto-curate threshold (Table S4, Tab “All detected proteins”, cells highlighted in yellow or red). For further comparisons, these values could not just be left out but were either adopted or replaced by zero using the following rules: A) If all three replicates within a group were only quantified with yellow or red auto-curate thresholds; all these values were set to zero. B) If two out of three replicates were quantified with a yellow or red auto-curate threshold; these values were set to zero, if the third value was also zero, or adopted if the third value was quantified with a green auto-curate threshold. C) If just one of the three replicates was quantified with a yellow or red auto-curate threshold, this value was adopted if at least one other replicate was quantified with a green threshold, or set to zero if both other values were also zero. The adapted cells were highlighted in light red or light yellow (Table S4, Tab “Proteins for evaluation”).

2) Finally, after this correction, all proteins that were quantified only in a single out of the twelve total samples (361 cases) were excluded. The final list of quantified proteins contained 1003 proteins (767 brain, day 0; 758 brain day 8; 253 HGs day 0, 679 HG day 8). With these proteins a Venn diagram was generated using VENNY 2.1 to illustrate tissue and time dependent changes (Oliveros, 2007).

Database adaptation for quantification

Obtained masses of all samples were initially reconciled against the reference protein sequences from A. mellifera (Amel_4.5, https://www.ncbi.nlm.nih.gov/assembly/GCA_000002195.1) yielding in a total of 1734 identified proteins and protein isoforms. However, exact quantification struggled as some proteins were automatically eliminated or misallocated by the program due to the following two reasons: i) for some genes different transcript/protein isoforms are annotated which result eventually in exactly the same proteins regarding amino acid sequence but are recorded as different proteins with different accession numbers in the NCBI database (e.g. Hbg 3 alpha-glucosidase, Gene ID 406131, 7 protein isoforms (XP_016767971.1, XP_016767969.1, XP_016767968.1, XP_006560870.1, XP_006560869.1, XP_006560868.1 & NP_001011608.1)). ii) although encoded by different genes some resulting proteins show an amino acid sequence identity of more than 95% (e.g. actin related protein 1, actin clone 205-like, actin clone 403-like and actin muscle like; Gene IDs: 406122, 551176, 552637 and 410075) leading again to quantification problems as an identified peptide cannot be proteotypically assigned to a specific protein and is thus neglected. Therefore, we constructed our own quantification reference database (Table S3) based on the 1734 proteins originally identified.

For this FASTA, each of the 1734 identified protein accession numbers was entered into the NCBI protein database (https://www.ncbi.nlm.nih.gov/protein/) and the corresponding gene was identified (Table S3, Tab “Final gene list”, Column A). Thereby, we identified a total of 1510 genes of which 871 (57.7%) were transcribed and translated into a single protein isoform and 639 (42.3%) genes resulted in more than one isoform (2 isoforms – 281 genes, 3 isoforms – 121 genes, 4 isoforms – 67 genes, 5 isoforms – 52 genes, 6 isoforms – 31 genes, 7 isoforms – 25 genes, 8 isoforms – 10 genes, 9 isoforms – 9 genes, 10 isoforms – 6 genes, >10 isoforms – 37 genes) (Table S3, Tab “Final gene list”, Column D). Of these “multi protein isoform genes”, 148 (23.16%) resulted eventually in the very same protein (Table S3, Tab “Final gene list”, Column E labelled “same”). 474 (74.18%) resulted indeed in different isoforms but with only slight differences in the amino acid sequence, e.g. differences in the N- and/or C-terminal amino acids (Table S3, Tab “Final gene list”, Column E labelled “iso”). In these cases, only the part of the isoforms that was identical between all isoforms was integrated into the database and labelled >GeneID_allXiso (Table S3, Tab “New database for mass spec”, Column A). Only in very few cases (17 of 639 genes – 2.66%) different annotated isoforms resulted in very different proteins as diverse exons of the very same gene were transcribed and translated (Table S3, Tab “Final gene list”, Column E labelled “realiso”). In these cases, all isoforms were added to the new database and named >GeneID_isoX (Table S3, Tab “New database for mass spec”, Column A).
Finally, all proteins of which additional sequence similarities were expected, e.g. MRJP1-9 or hexamerin 70a-c (Table S3, Tab “New database for mass spec”, Column B highlighted in blue) were again aligned using Clustal Omega 1.2.4 (Sievers et al., 2011) of the EMBL-EBI server. If identical trypsin-digested peptides were found, either non unique peptides were deleted for the database to guarantee an unambiguous assignment of the protein (Table S3, Tab “Final gene list”, Column H “non unique peptides deleted”) or if too many identical peptides were found, the proteins in question were combined for the database (Table S3, Tab “Final gene list”, Column H “combined with X for database”) and labelled as >GeneID_GeneID. In case of elongation factor 1-alpha (GeneIDs: 408385 & 544670), tubulin alpha (Gene IDs: 408388, 550827, 411519, 724291. 552766 & 412886) and tubulin beta (408782, 410559, 410994 & 410996) approximately the same amount of identical and unique peptides was identified between the isoforms. In these cases, multiple sequences were integrated into the database: one sequence including all identical peptides identifying all isoforms at once (>GeneID_GeneID_all), as well as one sequence identifying each specific isoform (>GeneID_unique). The final database included 1552 different proteins/protein isoforms (Table S3, Tab “New database for mass spec”). All mass spectrometry data have been deposited to the ProteomeXchange Consortium (http://proteomecentral.proteomexchange.org) via the PRIDE partner repository (Vizcaino et al., 2013) with the dataset identifier PXD012618.

2. Supplementary results

In general, protein amounts of the 20 most abundant proteins (Table S6, Table S4) were strongly influenced by protein, tissue and age (generalized linear model, GZLM; protein: W = 1244.64, df = 53, $P < 0.001$; age: W = 218.83, df = 1, $P < 0.001$; tissue: W = 499.54, df = 1, $P < 0.001$). An interaction was found between age and tissue (W = 264.10, df = 1, $P < 0.001$) which was primarily attributed to the increasing protein amounts in the hypopharyngeal glands (HGs) from day 0 (3.4 ± 4.4 fmol) to day 8 (15.3 ± 16.1 fmol). In contrast to that, protein amounts in the brain were similar between day 0 (19.6 ± 16.2 fmol) and day 8 (17.4 ± 15.4 fmol). Other interactions were found between age and protein (W = 601.56, df = 53, $P < 0.001$) as well as tissue and protein (W = 1040.43, df = 53, $P < 0.001$) as

many proteins were generally higher on day 8 compared to day 0 or in the brain compared to the HGs (Figure S6).

At day eight in the HGs, ten out of the 20 most abundant proteins were proteins involved in protein synthesis and folding, again emphasizing the massive protein-synthesizing nature of the HGs (Table S6). The most abundant protein in the brain, at both days, was the cytoskeletal protein actin (day 0: 56.5 fmol; day 8: 52.8 fmol), which was already indicated by the conspicuous band migrating at 40 kDa in the SDS PA gels (Table S6, Figure S4). Concordant with this, the cytoskeletal protein tubulin β is the second and third most abundant protein at day zero and eight, respectively. Also tubulin α is found within the ten most abundant proteins.

3. Supplementary discussion

Proteins connected to energy metabolism were abundantly present in the brain at both days, day zero and day eight (Table S6). This incorporates proteins directly linked to ATP metabolism (ADP/ATP translocase, ATP synthase α and β, phosphate carrier protein, arginine kinase) as well as proteins involved in glycolysis (pyruvate kinase, fructose-bisphosphate-aldolase, glyceraldehyde-3-phosphate dehydrogenase) and the citrate cycle (malate dehydrogenase). Large amounts of ATP are needed, for instance, in excitable nerve cells to establish sodium/potassium gradients with the help of the Na^+/K^+-transport ATPase (Skou, 1957). Both ATPase subunits belong to the 20 most abundant proteins in the brain (≥ 20 fmol) and are barely detectable in the HGs (≤ 1.4 fmol). Arginine kinase plays an important role in cellular energy metabolism especially in invertebrates, via catalyzing the reversible transfer of a phosphoryl group from ATP to arginine, resulting in phosphoarginine and ADP (Ellington, 2001).

In the HGs at day eight, many of the most abundant proteins are involved in protein synthesis and folding (Table S6). Among them, elongation factor 1-alpha (EF1α) provides binding of elongator aminoacyl-tRNAs to ribosomes and occurs in many eukaryotes in at least two functional copies, which are often tissue specifically expressed (Negrutskii & El'skaya, 1998). The genome of *A. mellifera* contains two copies, F1 and F2, of EF1α (GeneIDs 408385 and 544670) (Danforth & Ji, 1998) and our data confirm tissue specificity for these copies. Whereas in the HGs the total amount of EF1α (day 0: 11.8 ± 7.4 fmol; day 8: 34.7 ± 19.7 fmol) is provided only by EF1α-F2 (day 0: 10.8 ± 3.2 fmol; day 8: 42.6 ± 22.0 fmol), in the brain EF1α-F1 (day 0: 9.7 ± 1.8 fmol; day 8: 3.5 ± 3.4 fmol) accounts for approximately 20-30% of total EF1α (day 0: 28.7 ± 3.5 fmol; day 8: 20.0 ± 12.9 fmol).

Another protein being most abundant in the HGs at day 8 is glucose oxidase (23.9 ± 9.6 fmol). This enzyme produces by the oxidation of glucose hydrogen peroxide and gluconic acid, which is found at a concentration of 0.6% in both, royal and worker jelly (Nye, Shuel, & Dixon, 1973). Furthermore, glucose oxidase is also present in honey where the produced hydrogen peroxide represents a large share of the antibacterial activity of honey (White, Subers, & Schepartz, 1963).

The hexamerins 70c and 110, were almost exclusively detected at day zero, except for slight amounts of hexamerin 70c in the HGs at day eight, and belong in the HGs at day zero to the 20 most abundant proteins. This is not surprising, as hexamerins are synthesized by honey bee larvae in large amounts into the hemolymph where they serve as amino acid storage for the nonfeeding pupal period (Burmester & Scheller, 1999). Thus, also after hatching of the adult bees, the hexamerins are still massively present until they are depleted.

The uncharacterized protein LOC412543 contains two farnesoic acid methyl transferase (FAMeT) domains (PFAM12248), and is among the 20 most abundant proteins in the HGs at day zero (4.0 fmol). Besides that, its expression is even higher in the brain at both days (day 0 and 8: 17.4 and 9.8 fmol, respectively). FAMeT is an enzyme involved in the

biosynthetic pathway of juvenile hormone catalyzing the production of methyl farnesoate from farnesoic acid (Vieira et al., 2008).

Tubulins are encoded by a multigene family (Cowan & Dudley, 1983) and in *A. mellifera* six tubulin α (Gene IDs: 408388, 411519, 412886, 550827, 552766, 724291) and four tubulin β (Gene IDs: 408782, 410559, 410994, 410996) genes are annotated. After adaptation of the database, we were able to quantify total tubulin α and β amounts by identical peptides as well as the amounts of specific isoforms by unique peptides (Tubulin α encoded by 408388 and 550827 could not be distinguished as the resulting proteins share 448 out of 450 amino acids.). In case of tubulin α, the genes 408388/550827 always supplied the total amount whereas none of the other four homologues were quantified. For tubulin β, in the HGs at both days and in the brain at day 8 the gene 408782 provided the total tubulin β amount. In the brain at day 0, besides 408782, also 410994 accounts for 10-90% of total tubulin β dependent on the sample.

Another protein of the cytoskeleton, actin, is highly abundant in all samples. In a typical eukaryotic cell actin is usually considered to be the most abundant protein (Lodish et al., 2001). In addition, abundant actin filaments were found in the mushroom bodies of honey bee brains (Ganeshina, Erdmann, Tiberi, Vorobyev, & Menzel, 2012) which might explain the very high quantities of actin in the brain at both days (56.5 and 52.8 fmol, respectively). It was shown that the depolymerization of these actin filaments enhances associative olfactory memory in honey bees (Ganeshina et al., 2012).

4. Supplementary figures

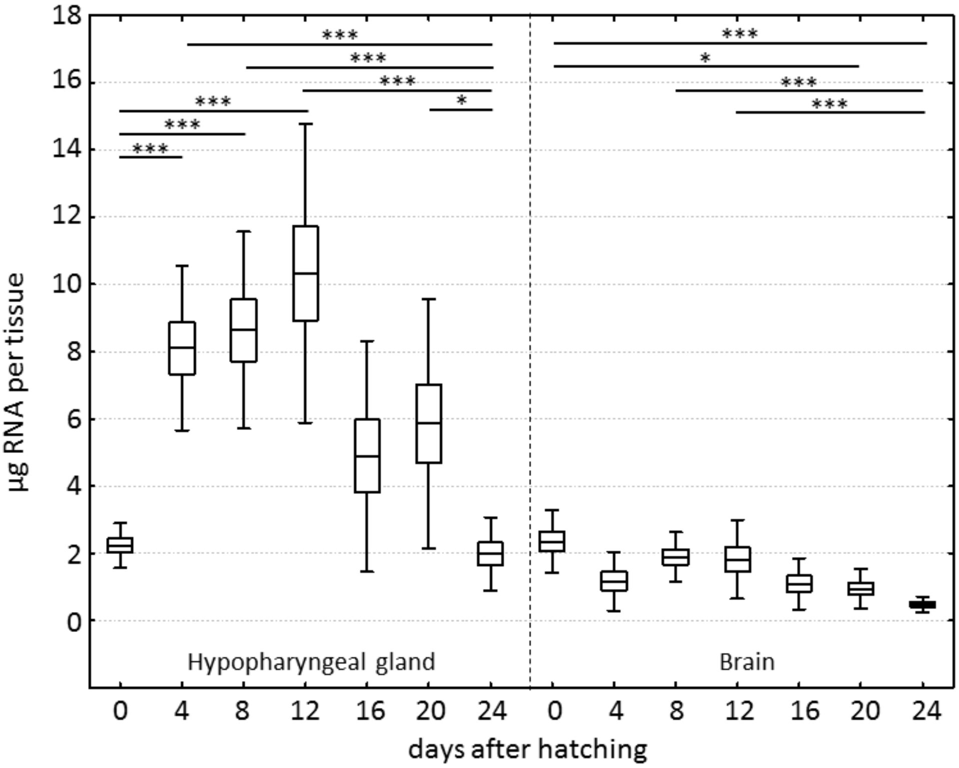

Figure S1: Total RNA isolated per hypopharyngeal gland pair or per brain per honey bee (n = 10 per time point). Boxes show means ± standard errors (SE) and whiskers show standard deviations (SD). Statistics have been performed using a full factorial ANOVA. Total RNA amount differs dependent on tissue and age of the worker bees (age: F = 15.51, df = 6, $P < 0.001$; tissue: F = 205.08, df = 1, $P < 0.001$; age × tissue: F = 6.63, df = 6, $P < 0.001$) with post-hoc Bonferroni tests. Significant differences are indicated by asterisks (*** $P < 0.001$, * $P < 0.05$). Within the HGs, RNA amount increased strongly after hatching to a constant level from day 4 to 12 (3.6 to 4.6-fold, in comparison to day 0; $P < 0.001$), followed by a similarly strong decrease until day 24 (4.1 to 5.2-fold, day 24 vs. days 4-12; $P < 0.001$) to the same level as directly after hatching. Total brain RNA amount has been generally lower (< 2.36 µg per brain) than total HG RNA amount (1.98-10.33 µg per HG pair; means) and did not show any pronounced difference over time. Only brains of 24 days old worker bees had a lower RNA amount (2.0 to 5.0-fold) compared to younger bees (days 0, 8 and 12 compared to day 24; P < 0.001).

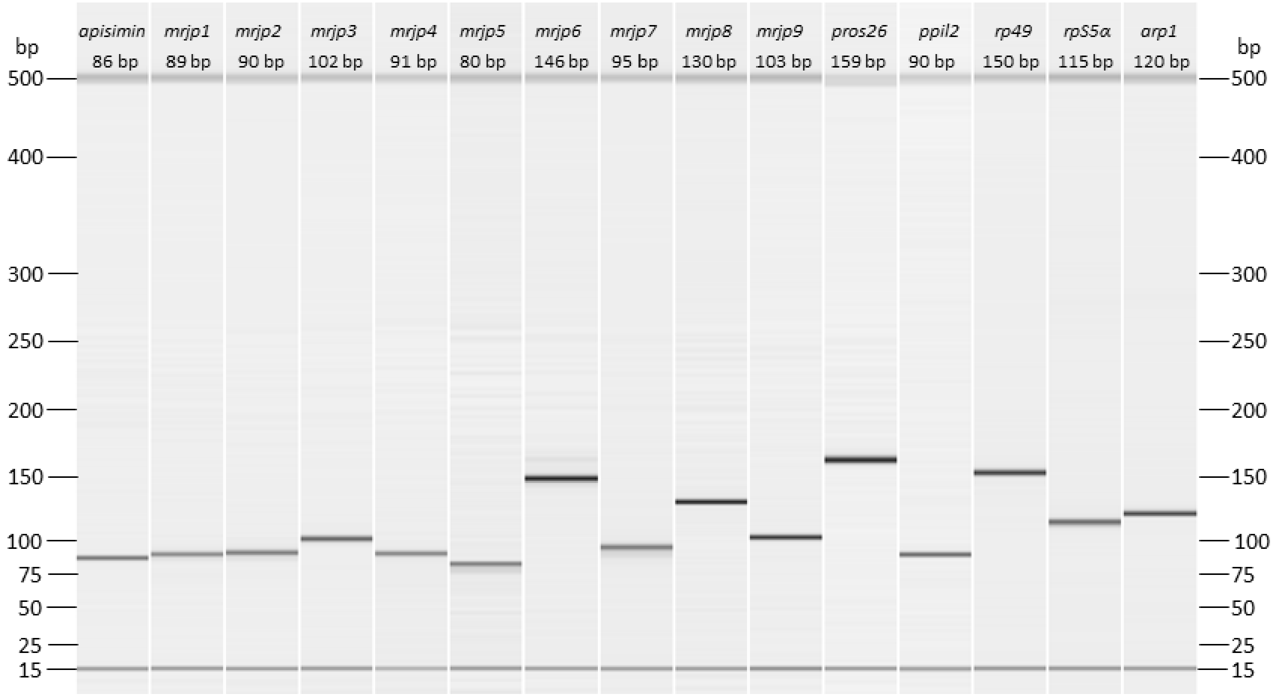

Figure S2: Analysis of qPCR product specificity.

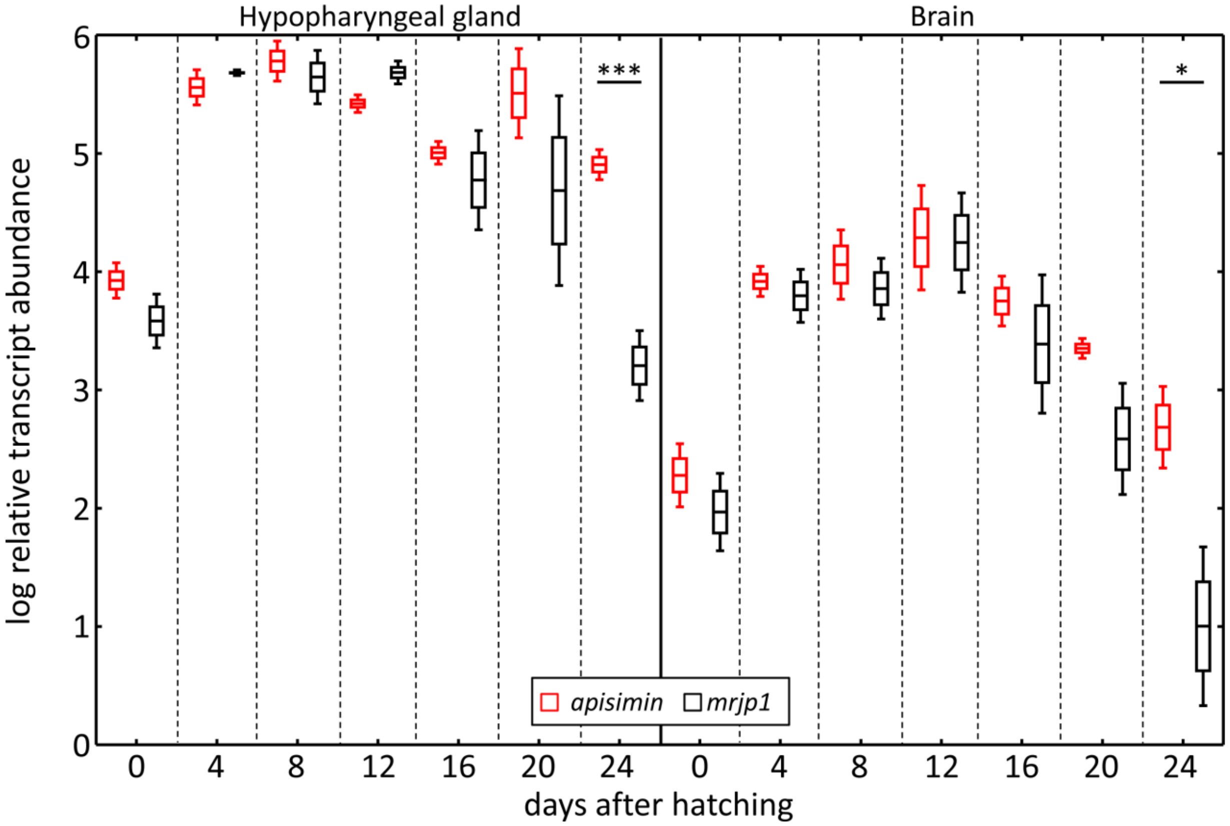

Figure S3: Relative transcript abundance (normalized gene expression standardized to RNA amount) of *apisimin* (red) and *mrjp1* in the hypopharyngeal glands and brains of worker honey bees. Boxes show means ± SE and whiskers show SD. Statistics have been performed using one-way ANOVA with post-hoc Bonferroni test. Significant differences are indicated by asterisks (*** $P < 0.001$, * $P < 0.05$).

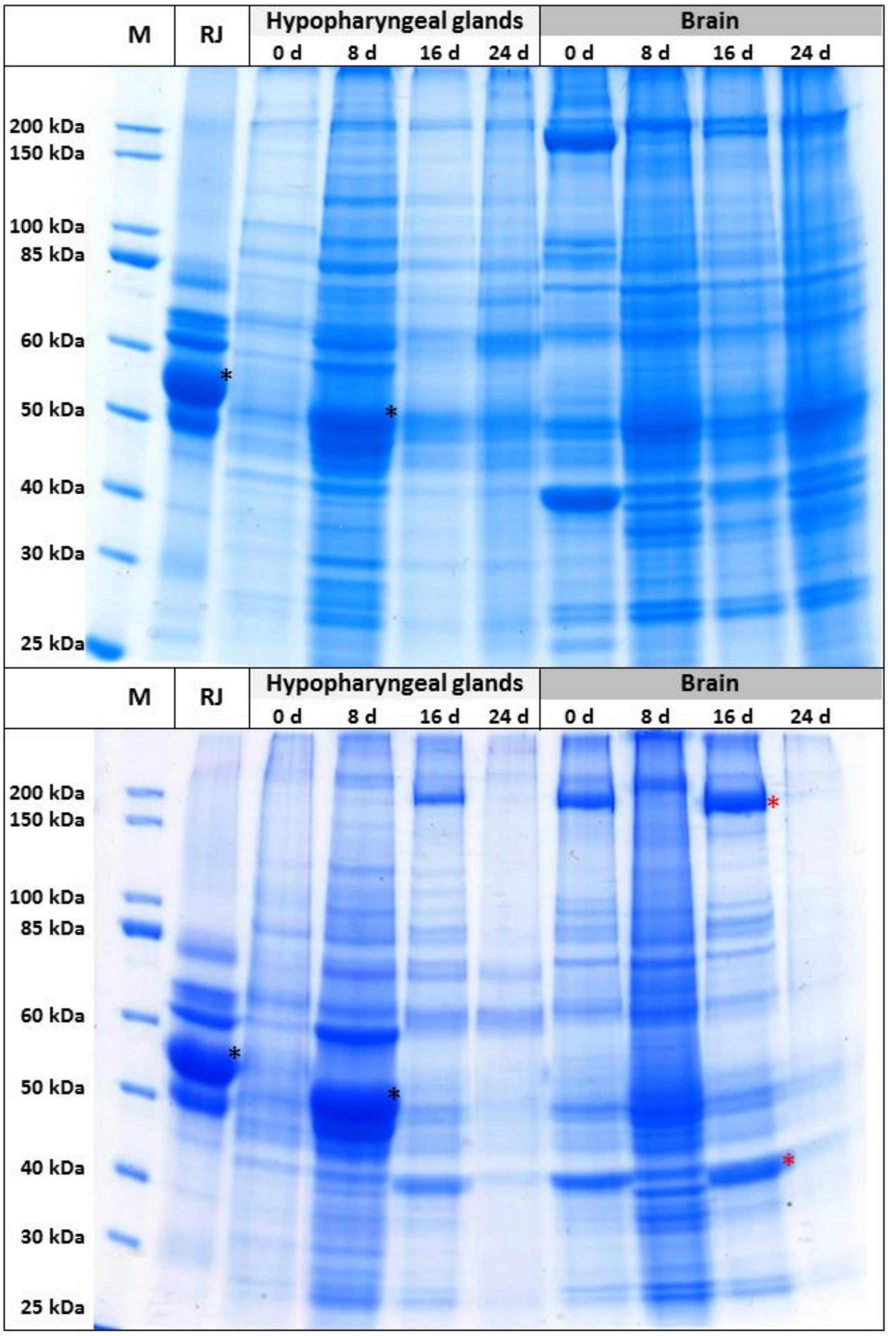

Figure S4: Two representative 8 % SDS polyacrylamide gels showing the proteins isolated per pair of hypopharyngeal glands and per brain per individual worker bee. M, unstained protein marker broad range; RJ, protein extract isolated from royal jelly, 0-24 d, zero to 24 days old worker bee; *band corresponding to MRJP1 in RJ and in the hypopharyngeal glands at day 8; *bands that were cut out of the gel and analyzed via mass spectrometry.

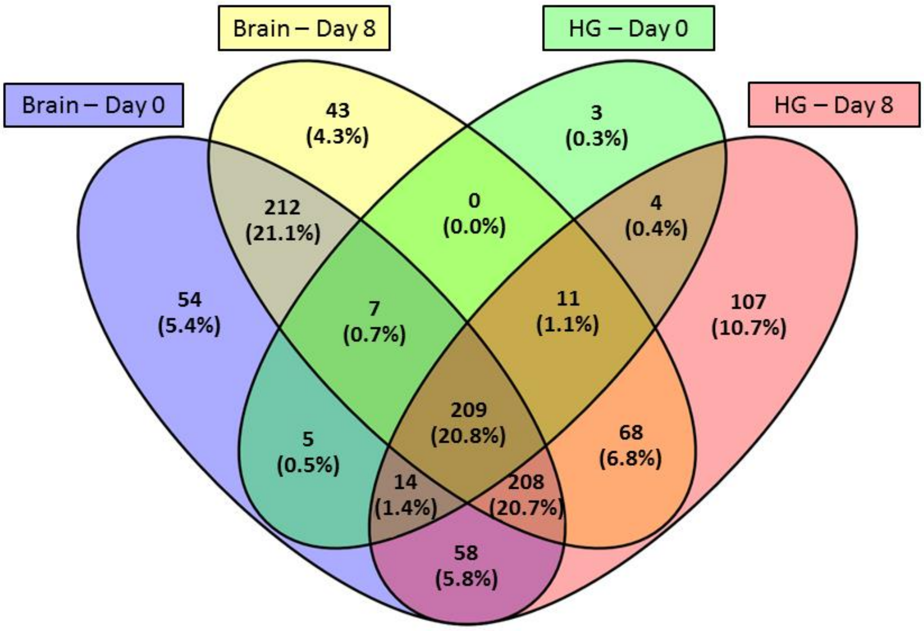

Figure S5: Venn diagram of unique and shared proteins within the hypopharyngeal glands and the brain of freshly hatched (day 0) or 8 days old worker honey bees. The color code is in accord with the color code in Table S4, column B.

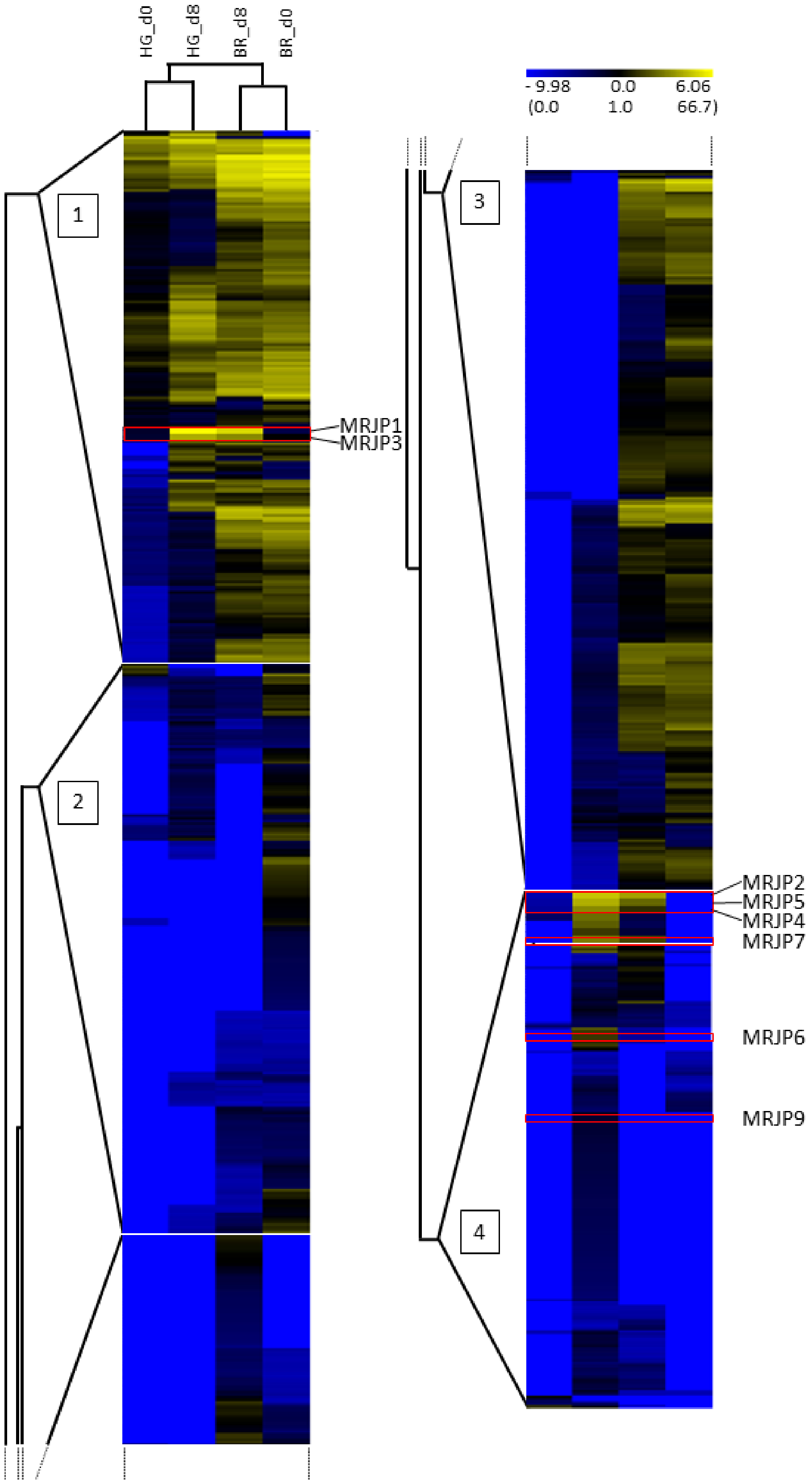

Figure S6: Heat map of mean protein amounts (fmol) in the hypopharyngeal glands (HGs) and brain (BR) of worker honey bees at day zero and eight. Protein amounts are represented as a color gradient from light yellow (highest) to deep blue (lowest). Values were log2 transformed (non-transformed valuea in brackets) and visualized using the MultiExperiment Viewer (MeV, mev.tm4.org) version 4.9. (n = 3 per day and tissue).

Table S1: Primer used for qPCR. *Mrjp, major royal jelly protein*; *pros26, proteasome subunit beta type-1*; *ppil2, peptidyl-prolyl cis-trans isomerase-like 2*; *arp1, actin related protein 1*; *rpS5α, ribosomal protein S5α*; *rp49, ribosomal protein 49*.

Name		Primer Sequence (5'-3')	Size (bp)	Product T_m (°C)	PCR efficiency	GenBank Gene ID	Reference
mrjp1	F	TGACATACATTACGAAGGAGTCCA	89	76.0	1.95	406090	Buttstedt et al., 2013
	R	ATCCGAAGAAGAGAACGCCA					
mrjp2	F	CGTCCAATACCAAGGATCCGAA	90	77.5	1.92	406091	Buttstedt et al., 2013
	R	ACAAGTCCGACGAAGAGGAC					
mrjp3	F	TGGACAGATGGCGTGATAAGAC	102	76.5	1.96	406121	Buttstedt et al., 2013
	R	GAGGTCCACCTTTGCCCTTT					
mrjp4	F	AGACAAAATATCGATGTCGTAGCTC	91	76.9	1.98	406133	Buttstedt et al., 2013
	R	TGCCAGATTGTGGAACGTTTT					
mrjp5	F	AGAAAATATGGATATGGTCGCTCAG	80	76.5	1.98	406116	Buttstedt et al., 2013
	R	ATTGTGGGAGATGCATCATTTTCA					
mrjp6	F	ACAAAATATGGATATGGTCGCTCAG	146	75.3	1.91	406149	Buttstedt et al., 2013
	R	TCTGCATTCTGTTACTTAAAGCCAA					
mrjp7	F	AGAAAATACCGATATGGTCGCC	95	76.0	1.98	409555	Buttstedt et al., 2013
	R	CAATAATGACGATATGTGGGAGCA					
mrjp8	F	TGGACTCAAGCATCGGCTAA	130	78.5	1.92	406067	Buttstedt et al., 2013
	R	TGGCAACCACTTCGATATTTCTT					
mrjp9	F	TCGGAAACTGGTGCTCTCTT	103	80.0	1.93	409873	Buttstedt et al., 2013
	R	TGGCGACTATTTCAATATTCCTTCT					
apisimin	F	TTCTGCGTAGCCATGTTGGT	86	80.0	1.95	406093	this study
	R	TGGGAAACGACATCCACGTT					
pros26	F	GCTGATAATGGAGGGAGTGTCA	159	77.5	1.92	411695	this study
	R	CCAACAACCTGAGCAACCCA					
ppil2	F	TTTAATGCGGCACACTATTCTACT	90	77.7	1.91	550966	Winkler et al., 2018
	R	AACTGCTGCTTGATGTGTAGTTTC					
arp1	F	TTGTATGCCAACACTGTCCTTT	120	78.5	n.d.	406122	Gregorc et al., 2012
	R	TGGCGCGATGATCTTAATTT					
rpS5α	F	AATTATTTGGTCGCTGGAATTG	115	74.9	n.d.	409728	Evans (2006)
	R	TAACGTCCAGCAGAATGTGGTA					
rp49	F	CGTCATATGTTGCCAACTGGT	150	77.0	n.d.	406099	Lourenço et al., 2008
	R	TTGAGCACGTTCAACAATGG					

Table S2: Relative transcript abundances for *mrjp1-7*, *9* and *apisimin* within the hypopharyngeal glands and the brain of worker honey bees from day 0 to day 24. *Mrjp, major royal jelly protein*. Each pool consists of three pairs of hypopharyngeal glands or three brains.

	day	pool	*mrjp1*	*mrjp2*	*mrjp3*	*mrjp4*	*mrjp5*	*mrjp6*	*mrjp7*	*mrjp9*	*apisimin*
hypopharyngeal glands	0	1	6328	249	921	724	243	954	65.2	0.96	8444
		2	3995	134	988	782	345	460	47.1	1.34	11908
		3	2232	59.8	313	382	196	781	23.1	0.96	5977
	4	1	462904	168200	59787	41823	22419	2981	48846	43.9	295404
		2	511406	123958	34969	24895	12414	3170	26242	10.5	536911
		3	469142	142236	47583	30970	11270	3688	34013	11.1	298510
	8	1	538104	156514	31798	19999	23647	6942	22965	22.7	717406
		2	245308	53634	14221	10535	12914	8222	11069	30.1	385540
		3	653861	190503	39649	23534	19133	6005	36021	69.6	793220
	12	1	454438	205156	74724	32229	19109	2909	42157	268	232335
		2	618553	309823	114347	36642	21954	2269	58053	180	319807
		3	401265	149153	46523	20394	13050	2152	28961	155	246773
	16	1	20334	4209	146	552	4014	3229	707	12.8	96762
		2	132253	47476	15398	5807	9030	2386	9324	30.4	128620
		3	78110	29301	8299	3533	6045	1787	5712	37.9	83484
	20	1	73543	13134	582	1230	8430	10447	2251	26.0	407808
		2	6433	436	7.26	151	1381	7876	220	4.86	123501
		3	240452	69374	18415	8954	10693	8374	10825	41.8	666869
	24	1	877	31.4	0.41	7.33	249	1472	1.61	2.14	75178
		2	3353	392	5.75	46.9	796	1911	36.0	5.23	62323
		3	1411	46.6	0.37	6.89	290	1884	1.25	2.43	110873
brain	0	1	178	5.33	22.6	21.0	8.19	45.6	2.03	2.31	279
		2	111	3.20	8.94	17.4	6.14	8.81	1.58	0.92	262
		3	40.6	0.77	3.34	5.49	2.29	16.2	0.41	0.41	93.6
	4	1	10864	3699	1100	865	361	54.0	1025	0.84	11163
		2	3906	953	200	238	130	31.0	276	0.21	6238
		3	5748	1432	339	444	167	70.4	464	0.68	8156
	8	1	3986	955	130	186	162	53.5	232	0.35	6040
		2	13005	2050	378	509	671	555	589	1.93	23188
		3	7173	1778	234	362	263	95.1	573	1.30	10819
	12	1	19314	8017	1938	995	735	82.2	1492	8.35	17092
		2	6432	2538	753	366	309	35.8	621	2.80	7501
		3	44160	16770	2396	1471	1024	148	2654	11.4	56685
	16	1	519	116	2.58	10.4	82.3	95.8	14.5	0.43	3223
		2	5958	1632	333	172	303	119	306	1.30	7576
		3	4724	1774	293	161	357	166	291	2.13	7383
	20	1	285	46.8	1.86	5.64	28.3	50.9	10.4	0.20	1843
		2	1284	319	51.1	44.0	46.3	38.8	51.8	0.64	2270
		3	157	7.90	0.13	1.37	22.0	85.7	4.25	0.30	2712
	24	1	15.5	0.65	0.08	0.25	4.94	27.4	0.08	0.11	837
		2	36.2	3.68	0.04	0.62	8.57	26.6	0.47	0.14	695
		3	1.81	0.08	0.00	0.01	0.27	2.14	0.04	0.05	195

Table S5: Correlation matrix for *mrjp1-7, 9* and *apisimin* normalized gene expression data showing Spearman's ρ correlation coefficients. Non-significant correlations ($P > 0.05$) are shaded in light grey. Correlation coefficients indicating a very high correlation (≥ 0.90) are highlighted in red.

	apisimin	*mrjp1*	*mrjp2*	*mrjp3*	*mrjp4*	*mrjp5*	*mrjp6*	*mrjp7*
apisimin	-	-	-	-	-	-	-	-
mrjp1	0.767	-	-	-	-	-	-	-
mrjp2	0.723	0.980	-	-	-	-	-	-
mrjp3	0.556	0.929	0.922	-	-	-	-	-
mrjp4	0.644	0.959	0.935	0.977	-	-	-	-
mrjp5	0.855	0.931	0.927	0.816	0.864	-	-	-
mrjp6	0.825	0.476	0.411	0.277	0.384	0.655	-	-
mrjp7	0.681	0.976	0.988	0.940	0.953	0.901	0.366	-
mrjp9	0.751	0.797	0.814	0.717	0.729	0.896	0.572	0.786

Table S6: Protein amounts (fmol; means ± SD) determined via quantitative mass spectrometry of the 20 most abundant proteins at day 0 and 8 within the hypopharyngeal glands and the brain of worker honey bees. Numbers in brackets show rankings of the proteins. The day and tissue at which the proteins were most abundant was highlighted in grey.

**Proteins**	**Hypopharyngeal glands**		**Brain**	
	**Day 0**	**Day 8**	**Day 0**	**Day 8**
40S rib prot S2*	1.3 ± 1.2	(17) 19.4 ± 13.9	4.4±0.7	4.9±1.6
40S rib prot S3*	2.3 ± 1.3	(19) 19.0 ± 14.9	6.3±1.1	5.2±3.8
40S rib prot S3a*	1.3 ± 1.1	(12) 21.3 ± 15.9	4.5±1.3	4.8±2.3
60S rib prot L3*	2.1 ± 1.5	(16) 19.6 ± 14.4	5.3 ±1.2	3.8 ±3.0
60S rib prot L4*	2.5 ± 1.8	(14) 20.6 ± 12.7	5.7±1.4	5.8±3.6
60S rib prot L9*	(16) 3.6 ± 1.7	(9) 22.7 ± 12.3	5.7±1.2	5.5±4.5
Actin	(3) 11.1 ± 3.0	(4) 30.2 ± 21.9	(1) 56.5 ± 7.5	(1) 52.8 ± 12.2
ADP/ATP translocase	2.7 ± 0.2	(6) 25.5 ± 20.0	(6) 40.5 ± 5.1	(6) 36.5 ± 12.7
Apidermin 3	(1) 21.7 ± 2.4	10.4 ± 4.9	2.8±2.5	1.1±1.1
Arg kinase	0.6 ± 0.5	7.2 ± 5.9	20.9 ± 5.7	(11) 24.3 ± 4.4
ATP synthase alpha	(14) 4.4 ± 0.6	17.7 ± 13.9	(4) 42.8 ± 9.9	(4) 38.0 ± 16.0
ATP synthase beta	(9) 5.6 ± 0.2	(10) 22.6 ± 16.8	(3) 52.3 ± 10.8	(2) 50.6 ± 18.7
EF1 alpha (both)*	(2) 11.8 ± 7.4	(2) 34.7 ± 19.7	(12) 28.7 ± 3.5	(19) 20.0 ± 12.9
specific F2 544670	10.8 ± 3.3	42.6 ± 22.0	20.9 ± 1.0	18.4 ± 15.4
specific F1 408385	0.1 ± 0.2	0	9.7 ± 1.8	3.5 ± 3.4
EF1 gamma*	(18) 3.0 ± 1.5	16.9 ± 11.2	8.7±2.5	5.4±4.1
Fructose-bisphosphate aldolase	0.3 ± 0.3	3.3 ± 3.2	8.1 ± 2.2	(17) 20.8 ± 12.1
Gap dehydrogenase 2	2.8 1.2	15.7 ± 8.9	(11) 32.7 ± 5.7	(8) 32.6 ± 15.7
Glucose oxidase	2.4 ± 3.0	(8) 23.9 ± 9.6	0	5.5±7.1
Guan nucl-bind protein SU beta	1.8 ± 0.7	(7) 24.8 ± 19.0	7.2±1.5	6.4±4.6
Hexamerin 70c	(19) 2.9 ± 1.9	1.1 ± 1.8	6.2±6.8	0
Hexamerin 110	(8) 6.2 ± 7.0	0	8.7±2.5	0
Hsc70-3*	(13) 4.5 ± 2.0	(11) 22.0 ± 18.6	(17) 23.9 ± 1.9	10.9 ± 5.8
Hsc70-4*	(4) 6.9 ± 1.7	(18) 19.4 ± 13.9	(7) 39.8 ± 5.0	(14) 22.4 ± 10.3
Hsc70-5*	(20) 2.9 ± 1.6	10.4 ± 7.6	17.0±2.2	10.0±4.6
Hsp60*	(5) 6.7 ± 2.4	14.3 ± 2.4	(16) 24.3 ± 5.0	16.0 ± 4.2
Hsp90*	0.9 ± 0.8	11.6 ± 10.6	(9) 34.6 ± 5.1	13.6 ± 8.5
Idgf4	(7) 6.5 ± 2.5	11.4 ± 5.8	(20) 21.0 ± 1.3	(20) 18.9 ± 9.7
LIMPETin	0	0	(14) 25.0 ± 15.5	14.2 ± 15.3
Malate dehydrogenase	0.9 ± 0.1	9.4 ± 5.5	17.7 ± 4.9	(16) 21.1 ± 11.9
MRJP1	(6) 6.6 ± 10.0	(1) 66.4 ± 5.8	1.6 ± 2.1	(5) 37.8 ± 9.8
MRJP2	0.2 ± 0.3	(3) 34.3 ± 22.8	0	14.7±2.4
MRJP3	(12) 4.6 ± 6.6	(5) 29.7 ± 24.4	1.3±1.2	11.1±6.6
MRJP5	0.5 ± 0.7	(13) 20.9 ± 10.2	0	9.1±10.0
Myosin heavy chain	0.1 ± 0.1	0	(5) 41.2 ± 18.7	(10) 27.6 ± 24.9
Na+/K+-transport ATPase alpha	0.2 ± 0.2	1.4 ± 1.6	(19) 21.3 ± 0.9	(12) 23.0 ± 10.6
Na+/K+-transport ATPase beta	0	0.4 ± 0.5	(18) 22.6 ± 3.8	(18) 20.0 ± 6.2
Paramyosin	0	0	(15) 24.7 ± 18.2	14.5 ± 15.3
Peptidyl-prolyl cis-trans isomerase*	2.4 ± 1.9	12.2 ± 10.3	(13) 27.0 ± 2.2	15.3 ± 8.0
Phosphate carrier protein	0.4 ± 0.3	11.6 ± 10.6	20.3 ± 2.7	(13) 22.6 ± 12.1
Porin	(10) 5.4 ± 1.8	11.9 ± 9.1	(10) 34.4 ± 8.8	(9) 32.3 ± 15.3
Pyr kinase	0.5 ± 0.1	5.6 ± 5.5	15.2 ± 2.2	(15) 21.3 ± 14.1
SN domain-cont protein 1	2.5 ± 1.8	(20) 18.8 ± 11.7	3.4±0.6	8.0±9.9
Transl elongation factor 2*	2.0 ± 1.2	(15) 20.6 ± 15.9	6.8±0.6	4.9±3.0
Tubulin alpha (all 6)	(17) 2.2 ± 0.8	6.1 ± 5.6	(8) 27.7 ± 8.8	(7) 32.6 ± 1.6
408388 + 550827 unique	2.9 ± 0.5	6.5 ± 6.0	35.7 ± 5.2	25.3 ± 3.8
Tubulin beta (all 4)	(11) 2.6 ± 0.2	9.0 ± 8.5	(2) 34.5 ± 10.3	(3) 29.2 ± 11.8
408782 unique	3.7 ± 1.1	8.3 ± 8.2	53.6 ± 14.5	38.6 ± 15.9
410994 unique	0	0	6.1 ± 9.3	0
unchar. protein LOC412543	(15) 4.0 ± 0.6	3.9 ± 3.5	17.4±2.0	9.8±4.7

*Proteins involved in protein synthesis and folding.

4. Additional supplementary references

Burmester, T., & Scheler, K. (1999). Ligands and receptors: Common theme in insect storage protein transport. *Naturwissenschaften*, 86, 468-474. doi: 10.1007/s001140050656

Cowan, N. J., & Dudley, L. (1983). Tubulin isotypes and the multigene tubulin families. *International Review of Cytology*, 85, 147-173. doi: 10.1016/S0074-7696(08)62372-4

Danforth, B. N., & Ji, S. (1998). Elongation factor-1 alpha occurs as two copies in bees: implications for phylogenetic analysis of EF-1 alpha sequences in insects. *Molecular Biology and Evolution*, 15, 225-235. doi: 10.1093/oxfordjournals.molbev.a025920

Ellington, W. R. (2001). Evolution and physiological roles of phosphagen systems. *Annual Reviews of Physiology*, 63, 289-325. doi: 10.1146/annurev.physiol.63.1.289

Ganeshina, O., Erdmann, J., Tiberi, S., Vorobyev, M., & Menzel, R. (2012). Depolymerization of actin facilitates memory formation in an insect. *Biology Letters*, 8, 1023-1027. doi: 10.1098/rsbl.2012.0784

Gregorc, A., Evans, J. D., Scharf, M., & Ellis, J. D. (2012). Gene expression in honey bee (*Apis mellifera*) larvae exposed to pesticides and Varroa mites (*Varroa destructor*). *Journal of Insect Physiology*, 58, 1042-1049. doi: 10.1016/j.jinsphys.2012.03.015

Lodish, H., Berk, A., Zipursky, S. L., Matsudaira, P., Baltimore, D., & Darnell, J. (2001). Section 18.1 The actin cytoskeleton. In *Molecular Cell Biology* (4th ed.). New York, NY: WH Freeman and Company.

Negrutskii, B. S., & El'skaya, A. V. (1998). Eukaryotic translation elongation factor 1 alpha: structure, expression, functions, and possible role in aminoacyl-tRNA channeling. *Progress in Nucleic Acid Research and Molecular Biology*, 60, 47-78. doi: 10.1016/S0079-6603(08)60889-2

Nye, M. J., Shuel, R. W., & Dixon, S. E. (1973) Gluconic acid in the food of larval honey bees. *Journal of Apicultural Research*, 12, 9-15. doi: 10.1080/00218839.1973.11099725

Skou, J. C. (1957). The influence of some cations on an adenosine triphosphatase from peripheral nerves. *Biochimica et Biophysica Acta*, 23, 394-401. doi: 10.1016/0006-3002(57)90343-8

Vieira, C. U., Bonetti, A. M., Simões, Z. L., Maranhão, A. Q., Costa, C. S., Costa, M. C., … Nunes, F. M. (2008). Farnesoic acid O-methyl transferase (FAMeT) isoforms: conserved traits and gene expression patterns related to caste differentiation in the stingless bee, *Melipona scutellaris*. *Archives of Insect Biochemistry and Physiology*, 67, 97-106. doi: 10.1002/arch.20224

White, J. W., Subers, M. H., & Schepartz, A. I. (1963). The identification of inhibine, the antibacterial factor in honey, as hydrogen peroxide and its origin in a honey glucose-oxidase system. *Biochimica et Biophysica Acta*, 73, 57-70. doi: 10.1016/0926-6569(63)90108-1
